# Mountain glacier extents at the Last Glacial Maximum

**DOI:** 10.1038/s41597-026-06841-z

**Published:** 2026-02-17

**Authors:** Augusto C. Lima, Helen E. Dulfer, Anna L. C. Hughes, Martin Margold, Iestyn Barr, Benjamin J. C. Laabs, Suzette G. A. Flantua

**Affiliations:** 1 Department of Biological Sciences, University of Bergen, Bergen, Norway; 2 School of Geography and Planning, University of Sheffield, Sheffield, S10 2TN UK; 3 Department of Geography, School of Environment, Education and Development, The University of Manchester, Manchester, UK; 4 Department of Physical Geography and Geoecology, Charles University, Prague, Czech Republic; 5 Department of Natural Sciences, Manchester Metropolitan University, Manchester, UK; 6 Department of Biological Sciences, North Dakota State University, Fargo, North Dakota USA; 7 Department of Biological Sciences, University of Bergen and Bjerknes Centre for Climate Research, Bergen, Norway; 8 Present Address: Discipline of Geography, School of Natural Sciences, Trinity College Dublin, University of Dublin. College Green, Dublin, 2 Ireland; 9 Present Address: U.S. Bureau of Reclamation, Denver, Colorado USA

**Keywords:** Palaeoclimate, Cryospheric science

## Abstract

Mountain regions experienced repeated glacial expansions and retreats during the Quaternary, shaping landscapes, ecosystems, and regional climates. While numerous reconstructions exist for individual mountain glaciers, global geodatabases remain scarce and rarely updated to reflect the latest observations. Here, we present GLACIMONTIS, a global geodatabase of maximum recorded areal extents of mountain glaciers at local Last Glacial Maximum, spanning 57-14 kyr BP. Our synthesis integrates reconstructions from 209 studies across 271 mountain ranges worldwide, compiling 15,014 individual glacier reconstructions, including 8,809 reconstructions compiled for the first time in a global geodatabase. Our work updates knowledge in 135 mountain ranges and highlights research gaps in 71 others. GLACIMONTIS represents the most comprehensive and up-to-date synthesis of mountain glacier areal extent at the global and local Last Glacial Maximum, providing spatial boundaries for refining climate-glacier modeling and delineating paleoecological reconstructions, and a framework for identifying regional research gaps. GLACIMONTIS advances Quaternary science by enhancing access to paleoglacier reconstructions and fostering interdisciplinary research in and across mountains worldwide.

## Background & Summary

Mountains hold crucial records for understanding Earth’s climate over time. A significant aspect of climate history is the legacy of glaciers, both in recent decades and in the geological past. Quaternary climate fluctuations caused repeated expansion and retreat of mountain glaciers and ice caps^1–3^, shaping landscapes through erosion and deposition^4–11^, and altering drainage networks^12^ and ecosystems^13^. Extensive mountain glaciation in the past had a localized cooling effect, influencing precipitation patterns and contributing to regional temperature regulation under various atmospheric conditions^14,15^. Undoubtably, glaciers have left an indelible mark on mountain regions, providing valuable insights into past climatic conditions, and shaping the environments we see today^16,17^.

The global Last Glacial Maximum (LGM, 26.5-19 ka)^18^ represents the last major culmination of global ice volume in the Late Pleistocene, in stark contrast to the warm climate conditions of the Holocene and interglacial intervals of the Quaternary Period. During the LGM, global mean temperatures were 6-4 °C cooler than present^19,20^. Recent noble gas paleothermometry further refines estimates, showing that low-latitude, low-altitude regions cooled by 5.9 ± 0.8 °C^21^. This widespread cooling drove major expansions of all components of the global cryosphere, although with distinct regional and local variations. The polar regions showed significant expansion of the Greenland and Antarctic ice sheets^22,23^. The Northern Hemisphere experienced growth of the Eurasian^24^ and the North American^23^ ice sheet complexes. Meanwhile, in the Southern Hemisphere, the Patagonian Ice Sheet covered much of southernmost South America^24^. This was matched by increased mountain glaciation across the globe with glaciers extending far beyond their current limits^25–31^.

Paleoglacier empirical reconstructions offer valuable insights into climate and mountain landscape development during the Quaternary^16,32^. They also support the validation of climate and glacier numerical models used for future climate projections^33^, and provide spatial boundaries for mountain ecosystems in the past^13,34,35^. Efforts to synthesize such data began with the global compilation of paleoglacier areal extents by Ehlers and Gibbard^36^ in 2004, which was last updated in 2011^37^ (https://booksite.elsevier.com/9780444534477/). While several regional compilations have followed for specific areas^38–44^, an up-to-date, comprehensive, and open-access global synthesis of glacier areal extents in mountain regions at and around the LGM remains absent. This stands in contrast to advancements in knowledge and available datasets of paleoglaciers and ice sheet areal extents over the last decade^24,45–48^.

Empirical reconstructions of former glacier areal extent have historically relied on the identification of distinctive glacial landforms, such as moraines, meltwater channels, ice-contact deltas, and other glacifluvial sediments^5,49,50^. These landforms are primarily identified through glacio-geomorphological field mapping, and can be guided by topographic maps, aerial photos, and other remote sensing imagery data and its products, such as digital elevation models (DEMs)^6,40,49^. While numerous studies acknowledge mapping glacial sediments and landforms known to delimit extents of glaciers^51^, not all of these studies reconstruct ice areas and thicknesses of past mountain glaciers. Additionally, mapping past ice margins is an interpretative process, that requires extensive knowledge and expertise, even when guided by glaciological principles and meticulous field mapping^52^.

To reconstruct paleoglacier area and thickness from mapped glacial landforms, flowline-based methods for reconstructing ice-surface profiles have been proposed^53–57^, and have since become a widely-adopted approach for mountain glacier reconstructions. These empirical approaches for geomorphological-based reconstructions calculate the ice surface area and ice thickness based on mapped glacial landforms and surface elevation data. Further, the local ice thickness, surface elevation and areal extent are interpolated for the whole glacier^55^. Meanwhile, advanced numerical paleoglaciological modeling^58^ and automated geomorphological mapping using artificial intelligence^59^ are opening new research paths for both paleoglacier reconstructions and glacial landform mapping.

These advancements in paleoglacier reconstruction, when coupled with dating techniques, have enabled more precise interpretations of the timing of glacial landforms and sequences. Such dating methods range from qualitative approaches, such as stratigraphic observations or relative dating (e.g., lichenometry, Schmidt Hammer, and tephrochronology)^60–62^, to quantitative radiometric techniques (e.g., radiocarbon dating, cosmogenic nuclide exposures dating, and luminescence dating)^40,63,64^. Although up-to-date global geodatabases of quantitative chronological ages of glacial landforms^65,66^ already exist, these databases do not provide information about the areal extent of the paleoglaciers.

Here, we present GLACIMONTIS, a global geodatabase of mountain glaciers extents at the local Last Glacial Maximum. Our primary objectives are to: (i) compile into a geodatabase the reconstructions of the last maximum areal extents of paleoglaciers in mountainous regions globally from published literature; (ii) record the original source’s findings on quantitative dating, interpreted Equilibrium-Line Altitude (ELA), and paleotemperature estimates of compiled reconstructions; and (iii) provide precise metadata linking each paleoglacier reconstruction to its original source.

To achieve our objectives, we developed two feature datasets of glacier reconstructions: (i) *Empirically Reconstructed Paleoglaciers*, which compile all reconstructions, organized by source and grouped by mountain range; and (ii) *Filtered Reconstructed Paleoglaciers*, a curated, ready-to-use output. The latter dataset merges reconstructions to create generalized glacier masks applicable for broad-scale applications of middle to low-resolution.

Overall, we aim for GLACIMONTIS to (i) support climate, cryosphere, and ecological modeling and validation workflows and (ii) identify variations, discrepancies, and research gaps in glacier reconstructions of past mountain glaciation by providing an accessible and cohesive global dataset of available paleoglacier reconstructions at their last glacial maxima.

### The concept of a global and local last glacial maximum

The term LGM refers to the period during Marine Isotope Stage 2 (MIS 2) marked by the peak in global terrestrial ice volume and the corresponding low in global eustatic sea-level, as indicated by a maximum in marine oxygen isotope (δ¹⁸O) values recorded in benthic foraminifera^67–72^. After the term was originally described by the CLIMAP Project Members^73^ and Cline *et al*.^74^, multiple studies worked on further constraining the LGM timing^18,67,68,75,76^. A widely accepted timing, based on minimum sea level during MIS 2, places the last global maximum in terrestrial ice volume between 26-19 ka^18^.

While the term LGM defines the global culmination of terrestrial ice volume, on local-to-regional scales, maximum ice extents of some ice sheets, ice-sheet sectors, and mountain glaciers were reached before or after the LGM^16,32,37,70,77^. To address this, the term ‘local Last Glacial Maximum’ (LLGM) has been used to differentiate local ice maxima that do not correspond to the accepted timing of the ‘global’ LGM^78–80^.

For GLACIMONTIS, our goal is to capture the maximum-achieved areal extent of mountain glaciers during the LGM and LLGM, accounting for both global trends and local variability. To achieve this, we focus on the period spanning the late MIS4 to early MIS1, as boundaries defined by Lisiecki and Raymo^72^. This timeframe (57–14 kyr BP) encompasses the global LGM and offers a more comprehensive representation of local and regional glacial maxima recorded across mountain ranges worldwide (i.e., LLGM). As GLACIMONTIS refers to the last maximum areal extent of paleoglaciers at the LGM and LLGM, we are not compiling continuous retreat and advance extents (e.g., deglacial extents of the former ice sheets) nor smaller maximum ice extent assigned to other glacial events (e.g., Henrich Stadial 1) during the timeframe.

The selection of the timeframe (57–14 kyr BP) is not intended to redefine the LGM chronozone, nor to propose a new LGM chronology, but to accommodate the diachronous nature of the last maximum areal extent of mountain paleoglaciers. This approach has been proposed, discussed and applied in previous global geodatabases of glacier reconstructions at the LGM and LLGM^36,37,42,44^. By adopting this broader temporal framework, GLACIMONTIS includes reconstructions even when precise dating is unavailable for a particular glacier or regions (e.g., relative dating techniques rather than an LGM age established by quantitative dating) ensuring a more complete synthesis of the last maximum areal extent of mountain glaciation. Henceforth, we use the term ‘LLGM’ to denote the last local glacial maximum across individual mountain ranges, whether or not it coincides with the global LGM, as mountain ranges often achieved their maximum extent locally and asynchronously, even during the global LGM.

## Methods

### Literature review

We conducted a comprehensive literature review to compile the last maximum areal extents of mountain glaciers within our defined time interval. The process began with a foundational reference collection collected by all co-authors, covering key publications from all continents and major mountain ranges. We complemented this baseline collection with systematic searches in *Google Scholar* and *Zenodo*, prioritizing empirically based reconstructions of glacier areal extents. All data incorporated into GLACIMONTIS originate either from peer-reviewed publications or from datasets released by governmental agencies.

The review involved two stages: (1) an initial search to identify relevant publications, and (2) the application of inclusion/exclusion criteria to refine the results (see details in Selection Criteria). Using our baseline collection as a starting point, we applied the following search terms in *Google Scholar* and *Zenodo*: (“Glacier Reconstruction” OR “Glacial History” OR “Maximum Ice Extent” OR “Maximum Glacial Advance”) AND (“LGM” OR “Last Glacial Maximum” OR “Late Pleistocene” OR “Last Glaciation” OR “Last Glacial Cycle”). Given the high number of results returned by *Google Scholar*, we used the first 10 pages as an initial selection while cross-checking with key synthesis publications and previous databases^28,29,36,37,42,77,80,81^ to ensure broad coverage. This combination of methods aimed to capture the full scope of available reconstructions while minimizing selection bias.

The suite of key global and regional synthesis publications was used to identify areas with missed reconstructions and guide further synthesis efforts per continent or mountain range, where mountain glaciation has been reported during 57-14 kyr BP, but reconstructions were missing in the GLACIMONTIS. To address such potential gaps, we created virtual libraries of collected publications using *ResearchRabbit* (https://researchrabbitapp.com/) to identify additional literature based on citation connectivity per continent and mountain range. A total of 526 publications were compiled and further assessed for their relevance in providing reconstructions of the last maximum areal extent of glaciers.

### Selection criteria

**Fig. 1:**
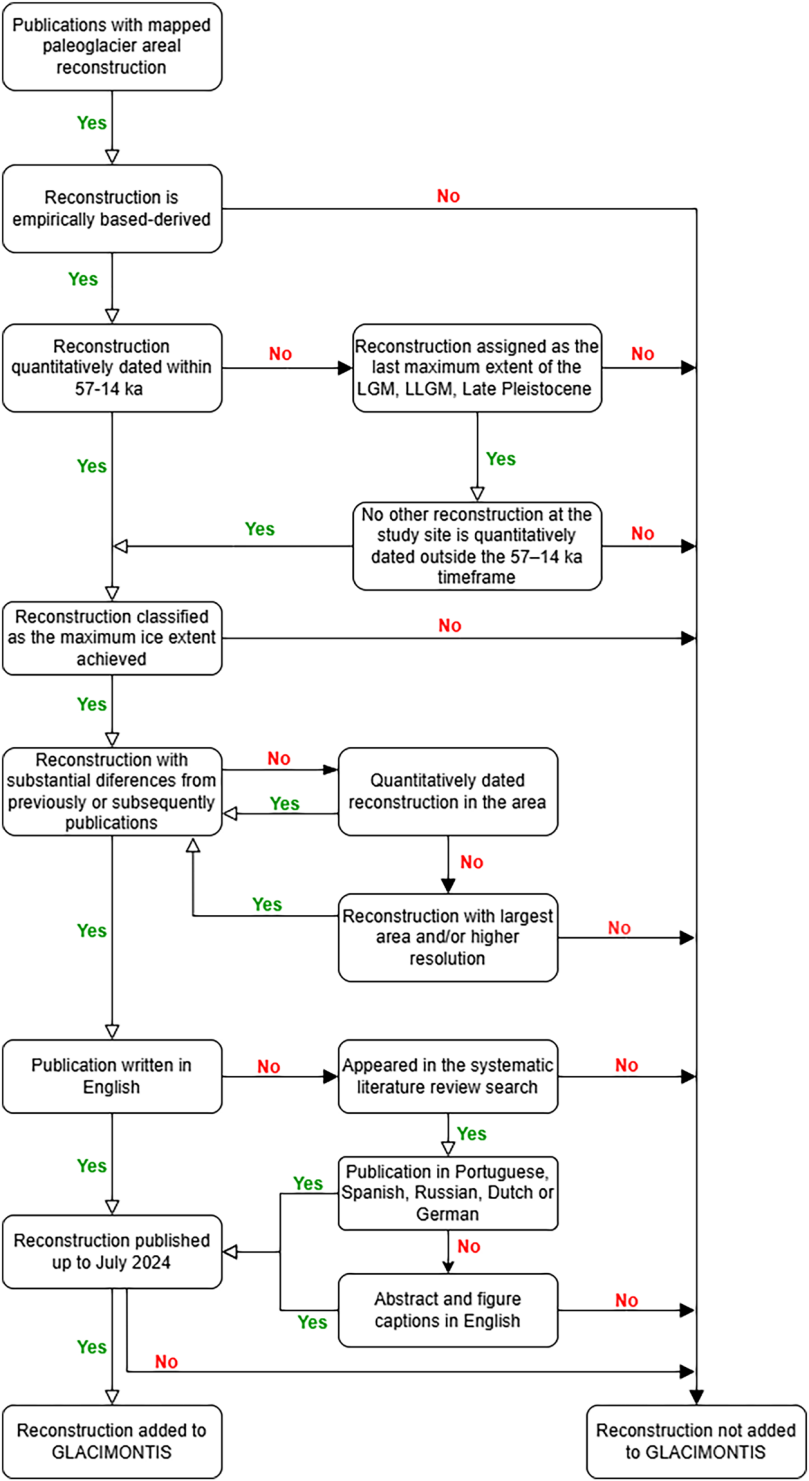
Inclusion and exclusion criteria applied in the GLACIMONTIS literature review. Only mapped paleoglacier reconstructions dated within 57–14 ka (or assigned as the last maximum extent of the LGM, LLGM or Late Pleistocene), and meeting quality, duplication, language, and publication date criteria (before July 2024) were retained.

Inclusion and exclusion criteria were applied to further refine the initial literature review (Fig. 1). The flowchart illustrates the sequential decision rules used to determine whether a reconstruction was retained in GLACIMONTIS.

First, publications had to include original paleoglacier reconstructions presented spatially as a map delineating the glacier’s areal extent. In cases of overlapping reconstructions from different publications but without substantial differences, the version with the largest reconstructed area and/or higher resolution was selected. In the cases where the exact same reconstruction was presented in different publications, we selected the first published reconstruction. Second, the timing of the reconstruction had to fall within the MIS boundaries 4/3 (57 kyr BP) to 2/1 (14 kyr BP)^72^ or be assigned by the authors to the LGM, LLGM, or as the last maximum ice extent achieved during the Late Pleistocene (if quantitative dating was unavailable). In the latter case, some reconstructions lacking chronological constraints may have been excluded from our dataset if subsequent paleoglacier reconstruction with quantitative chronological dating placed them outside our selected time range. Third, only reconstructions classified as the maximum ice extent of glaciers, ice caps, or ice fields were included, while areas covered by continental ice sheets during MIS 2 (e.g., East and West Antarctic Ice Sheets, Greenland Ice Sheet, Icelandic Ice Sheet, Eurasian and North American ice sheet complexes, and the Patagonian Ice Sheet) were excluded, including the advance of glaciers in mountain regions before they coalesce to form an ice-sheet and their deglacial retreat following the retreat of the ice sheet.

Although the search primarily targeted English-language publications, non-English works were included if they (1) appeared in the literature search, (2) provided abstracts and figures with English descriptions, or (3) were in a language spoken by one of the authors (Portuguese, Spanish, Russian, Dutch, German). Only outputs published up to July 2024 were included, making this the census date for this version of GLACIMONTIS. Ultimately, 209 publications meeting these criteria were included in the final literature review compilation.

### Access to geospatial features

The focus of GLACIMONTIS was to compile reconstructed areal extents of mountain glaciers that represent the last maximum extent during the LLGM. As the presence of a reconstructed areal extent was a selection criterion in our literature review, publications only presenting glacial landforms, such as moraines and/or chronological information, but lacking a mapped outline of the reconstructed paleoglacier were not included. Therefore, the lack of spatially reconstructed glaciers in any specific mountain region is not necessarily indicative of a lack of paleo-glaciological research or evidence of past glaciation (see *Glaciated Mountain Ranges Overview*).

Spatial reconstructions of paleoglaciers were obtained from a combination of sources. When available, data were downloaded directly from open-access repositories (e.g., *Zenodo*) or from supplementary materials of journal articles. For reconstructions not openly available, we requested data from the corresponding authors. If requests went unanswered or data were unavailable in digital format, reconstructions were manually digitized in ESRI ArcGIS Pro 3.2.0^82^ from published figures to obtain a derivative product. In cases where authors explicitly declined to have their reconstructions included in GLACIMONTIS, we respected their decision.

Not all reconstructions added from Ehlers and Gibbard^36^ and Ehlers *et al*.^37^ were incorporated to GLACIMONTIS. The reconstructions in Tien Shan (China), Romania, Falkland Islands, Mexico, Lesotho and Afghanistan were only present as figures in the accompanying book chapters, not in the accompanying geodatabase. We also noted that, the extent of glaciation in New Zealand shows differences between the digitized reconstructions included in the database and those presented in the original associated book chapters (we included both). In addition, the reconstructions within the geodatabase from Jan Mayen, Corsica, Serbia, Faroe Island, Balkai-Stanovoy (Russia) and Sikhote-Alin (Russia) were not directly linked to any of the region-specific book chapters, thus those reconstructions were referenced to the introduction chapter of these edited volumes, as well as the geodatabase. Finally, the Bini^83^ reconstruction for the Swiss Alps had to be re-digitized, due to incompatibility of their line feature format with our polygon-based geodatabase.

Manual digitization of printed maps introduces potential uncertainties due to georeferencing errors, the absence of geographic coordinate systems, or variation in image quality. Common challenges include: (i) coarse or inconsistent resolution of published maps, (ii) missing coordinate grids, map projections, or geographic reference features (e.g., rivers, topographic basemaps, administrative boundaries), and (iii) ambiguous boundaries in figures (e.g., pixelated shading, generalized outlines). These issues may produce slight deviations between the derivative product and the original figure. Because the original spatial data are unavailable in such cases, accuracy cannot be quantitatively assessed.

Moreover, it is impossible to define a strict, uniform digitization method. The process varied depending on how figures were created and published, and it often requires subjective interpretation of terrain when geographic coordinates are missing. Even when coordinates were provided, not all publications specified the projection, datum, or spatial reference system used, further complicating reproducibility. In some cases, maps were also not printed at a consistent scale. As a result, digitization should be understood as an adaptive and interpretative step to obtain a derivative product with limitations, inherently tied to the quality and clarity of the original source material.

To maintain transparency, we synthesized and compiled all overlapping or coexisting reconstructions for a given region, when they presented sufficient uniqueness to warrant representation (see Selection criteria). Rather than a limitation, this diversity reflects the current state of research and enables users to evaluate alternative reconstructions. Users are encouraged to critically assess data resolution, provenance, and methodological background when selecting reconstructions for their own applications.

### Organization of metadata and findings

The source metadata (e.g., source publications, methods, and availability) and paleoglacier/paleoclimate information (e.g., chronology, modern and paleo Equilibrium Line Altitude (ELA), and paleoclimate) were first organized into the *GLACIMONTIS Worksheet*, later reproduced as standalone spreadsheets in the GLACIMONTIS geodatabase, and finally linked to the mapped paleoglacier reconstructions. The GLACIMONTIS worksheet is organized into two spreadsheets:

1. [Metadata Information Spreadsheet]: Summarizes source citation metadata, data acquisition and availability, and methodological details for each publication.
2. [Paleoglacier/paleoclimate Information Spreadsheet]: Records synthesized quantitative information for the paleoglacier reconstructions.

To ensure cross-referencing between tables, each publication was assigned a unique Metadata Identifier (MID). Individual paleoglacier reconstructions were catalogued using Paleoglacier Identifiers (PIDs). Reconstructions from the same MID that span multiple mountain ranges were grouped under a Geographic Identifier (GID), which standardizes records at the mountain-range scale.

### Metadata information spreadsheet

This spreadsheet documents the metadata and methodological details of each source publication used in GLACIMONTIS. The Metadata Information Spreadsheet describes the bibliographic, methodological, and data-access attributes systematically extracted from each publication (see more in Data Records). Together, these fields provide traceability of sources, transparency of methods, and information on the type and accessibility of paleoglacier reconstructions.

Each reference is assigned to a unique Metadata Identifier (MID), which links its records to the *Paleoglacier/Paleoclimate Information Spreadsheet*. This allows users to cross-check bibliographic, methodological, and reconstruction details across the GLACIMONTIS database and original sources.

### Paleoglacier/paleoclimate information spreadsheet

This spreadsheet provides an overview of selected quantitative findings reported in the original publications. We synthesized attributes related to timing of deglaciation, equilibrium-line altitudes, and associated paleoclimate estimates (see more in Data Records).

Emphasis on paleoclimate reconstruction-derived attributes was given as they are variables most frequently used to infer past climate conditions, and therefore directly relevant for paleoclimate syntheses. Recording dating chronology ensures traceability of temporal constrains and allows identification of potential inconsistencies or uncertainties across reconstructions.

Each record in the Paleoglacier/Paleoclimate Information Spreadsheet corresponds to a unique PID, representing the specific paleoglacier or set of paleoglaciers reconstructed in a study. For example, Lee *et al*.^84^ reported paleo ELA estimates for ten distinct paleoglaciers reconstructed in Laguna de las Huaringas (Tropical Andes). Each paleoglacier is recorded as a separate entry with its own PID, but all entries share the same MID. By contrast, if a publication only provided generalized regional findings, without paleoglacier-level details, the reconstructions are assigned the same PID. For example, Barrows *et al*.^85^ provided a general timing estimation for the overall glaciation in Tasmania and did not provide paleoclimate estimation for each individual reconstructed paleoglacier.

The GID further organizes reconstructions by geographic unit (i.e., mountain ranges), ensuring spatial consistency across the database and its synthesized information. Continuing the example of Lee *et al*.^84^: although 10 distinct PIDs are recorded, all fall within the same mountain range and are therefore assigned a common GID. Note that only paleoglacier reconstructions with the same MID can be assigned a common GID.

### Preprocessing and geodatabase construction

**Fig. 2:**
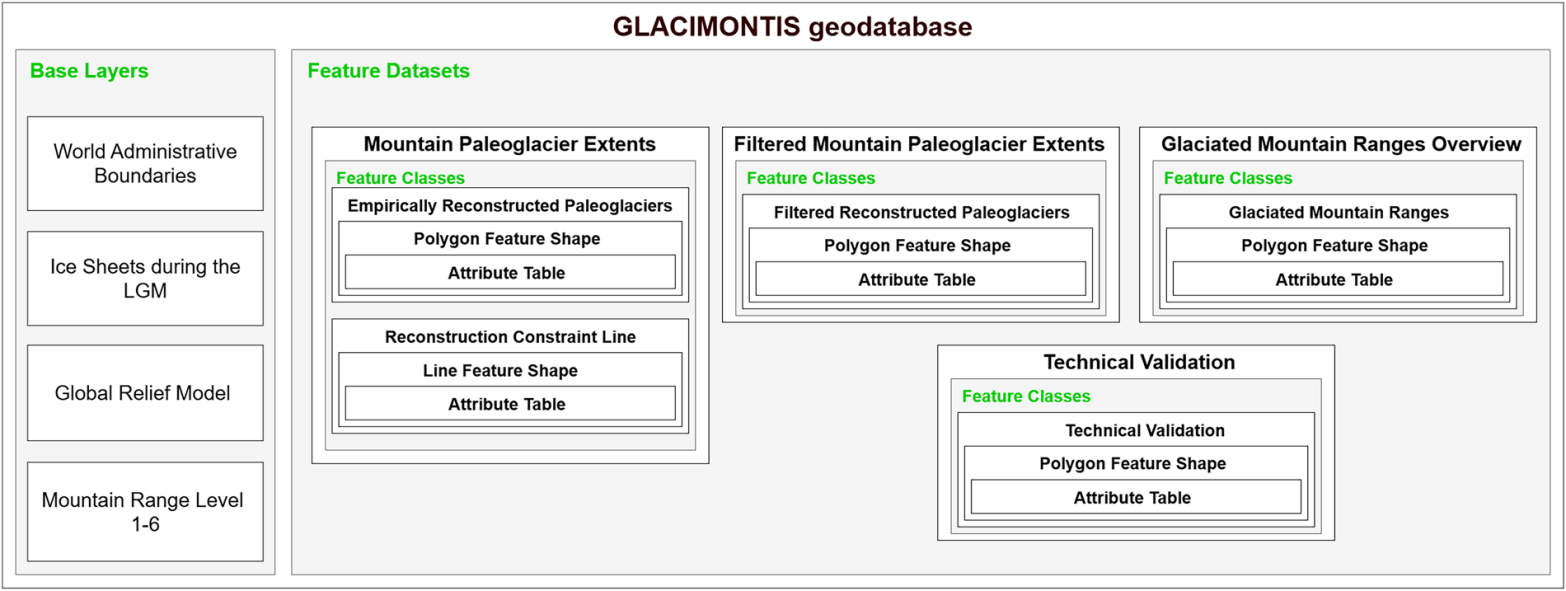
GLACIMONTIS structure with the Base Layers, Feature Datasets and corresponding feature classes and attribute tables. Base layer sources (see more in section GLACIMONTIS Geodatabase – base layers): World Administrative Boundaries^86^; Reconstructed Ice Sheets^22,45,81,87,88^; Global Relief Model^89^; Global Mountain Biodiversity Assessment^90,91^. A feature dataset is a container that groups together related feature classes. A feature class is a collection of spatial feature shapes (e.g., points, lines, or polygons) with a common set of attributes.

The GLACIMONTIS *geodatabase* refers to all features, layers, and tables presented in this work. The geodatabase consists of base layers and multiple feature datasets. Each feature dataset contains feature classes, represented as collections of spatial features linked to attribute tables. The feature datasets are Mountain Paleoglacier Extents, Filtered Mountain Paleoglacier Extents, Glaciated Mountain Ranges Overview, and Technical Validation (Fig. 2).

The base layers serve as the geographic and topographic framework for all feature datasets. The World Administrative Boundaries^86^ provide modern political borders for spatial reference. The Reconstructed Ice Sheets include outlines of the Laurentide^87,88^, Cordilleran^87,88^, Fennoscandian^87,88^, Patagonian^45,81^, Greenland^87,88^, Iceland^87,88^, and Antarctic^22^ ice sheets during the LGM, offering a large-scale cryospheric context. The Global Relief Model^89^ supplies a 30 arc-second continuous digital elevation and bathymetry background to situate paleoglacier extents within topographic settings. Finally, the Global Mountain Biodiversity Assessment (GMBA v2.0, https://www.earthenv.org/mountains)^90,91^ mountain ranges dataset provides a hierarchical delineation of mountain ranges, enabling consistent classification and regional comparisons across GLACIMONTIS.

All the paleoglacier reconstructed data presented in the GLACIMONTIS geodatabase are linked to the original references and DOI codes compiled in the *Metadata Information Spreadsheet*. When original sources already provided data in open-access format, archived in repositories or supplementary material, direct links for data download are also provided in the *Metadata Information Spreadsheet* (see full attribute list in Data records).

In the following subsections, we describe the construction of the *Mountain Paleoglacier Extents*, *Filtered Mountain Paleoglacier Extents*, and *Glaciated Mountain Ranges Overview* feature datasets, while the *Technical Validation* feature dataset is presented separately in its own section.

### Mountain paleoglacier extents

The *Mountain Paleoglacier Extents* is the primary feature dataset in the GLACIMONTIS geodatabase, serving as a unified spatial framework that consolidates all compiled reconstructions and their associated metadata for the last maximum areal extents of mountain glaciers during the LLGM. This dataset contains two feature classes:

1. *Empirically Reconstructed Paleoglaciers*: polygon feature shapes of reconstructed glaciers compiled from published landform-ice surface profile models or from expert interpretation of glacial records. The landform-ice surface profile models uses mapped glacial landforms (e.g., terminal moraines) that represent past glacier boundaries, the surrounding topography (e.g., digital elevation models) and catchment area to derive paleo-ice surface profiles from glaciological principles^55^, using GIS tools such as GlaRe^56^, VOLTA^53^, REVOLTA^54^, and PalaeoIce^55^, or even flowline models represented in spreadsheets^92^. In the second case, reconstructions derived from expert interpretations of glacial landforms often combine extensive field-based geomorphological mapping and in-depth understanding of glacial dynamics.
2. *Reconstruction Constraint Line*: created line features that delineate the geographical limits within which reconstructed glaciers are constrained. This feature class is particularly useful when reconstructions are incomplete due to factors such as political boundaries^93^, regional focus^38,94^, or specific study areas^95–99^. These boundaries help differentiate non-reconstructed areas from genuinely non-glaciated regions, while also identifying incomplete reconstructions.

This feature dataset is a multi-resolution product that preserves the spatial resolution and boundaries of the original glacier reconstructions. Reconstructions are preserved side by side to reflect the diversity of available paleoglacier outlines rather than merged into a single outline. This means that rescaling, smoothing, quality control, or additional editing were not applied. Preprocessing was limited to file format conversion (e.g., from*.nc* to*.shp*, or from line to polygon feature shape) conducted in ESRI ArcGIS Pro 3.2.0^82^. For details on georeferencing and digitization uncertainties, see *Access to geospatial features*.

**Fig. 3:**
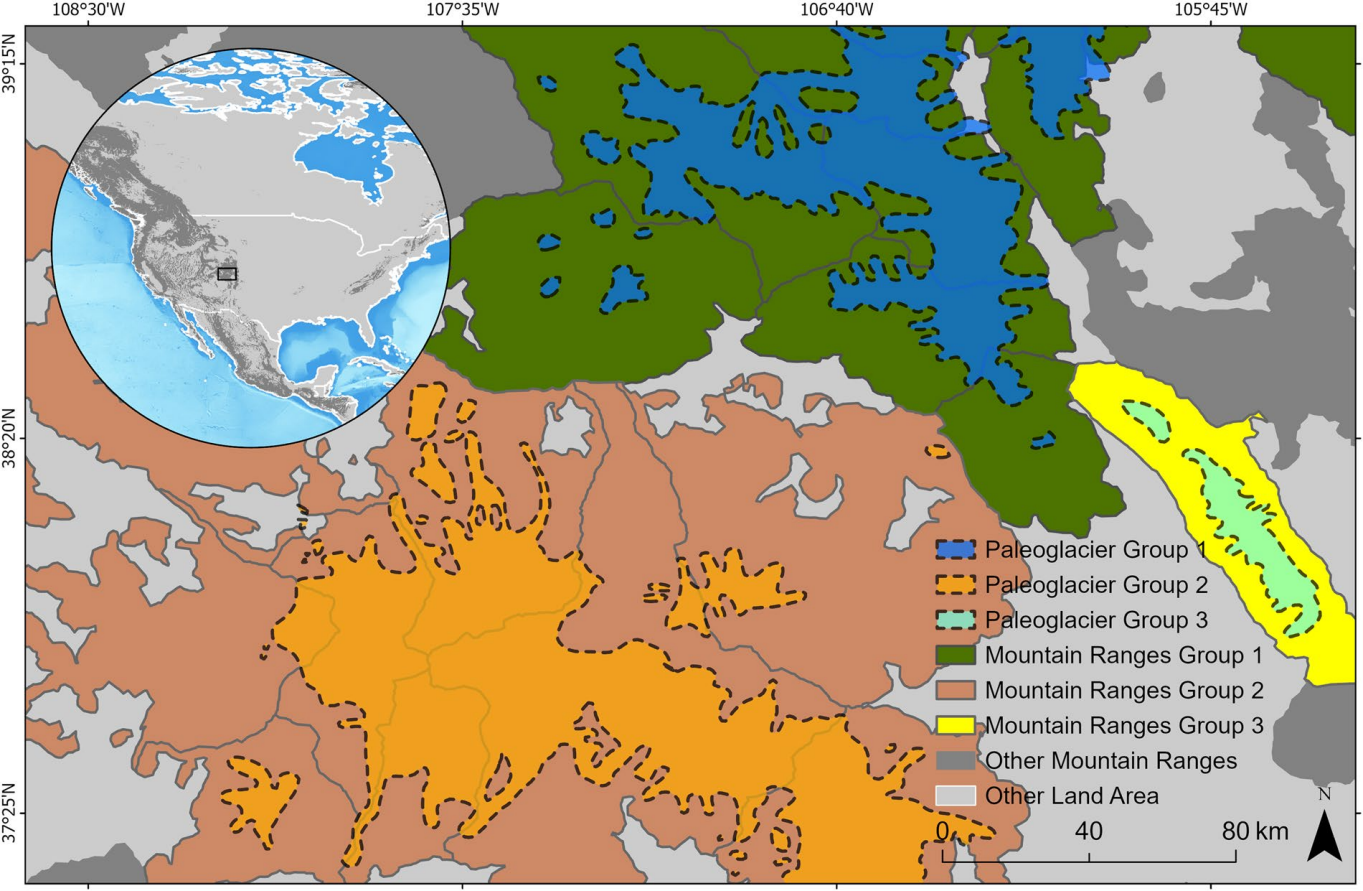
Assignment of paleoglacier reconstructions to paleoglacier groups based on mountain range classification. Illustration of how individual paleoglacier polygons are grouped within the hierarchical mountain range framework of GMBA v2.0^90,91^. Polygons that fall within the same mountain range are grouped into a single glacier group as multipart features. For example, Paleoglacier Group 1 is composed of several individual paleoglaciers that span across different mountain ranges (level 6), in which they share a common mountain range (level 6) at some point in space. However, Paleoglacier Group 1 is separated from Paleoglacier Group 2 and Paleoglacier Group 3 because there is not one single mountain range (level 6) that these paleoglaciers share. The grouping only happens within reconstructions from the same source, as the example showing reconstructions from Dahms^103^ in the Southern Rocky Mountains, western USA.

To ensure global consistency for multi-scale and comparative analyses, we overlaid all reconstructions with the hierarchical mountain classification system of the GMBA v2.0^90,91^. Mountain range names (Levels 1–6) were automatically assigned to each reconstructed polygon through spatial intersection in ESRI ArcGIS Pro 3.2.0^82^. Further, the polygon feature shapes from the same source (i.e., common MID) were grouped into multipart features if they shared the same highest-level mountain range classification based on GMBA v2.0^90,91^ (i.e., they occur within the same (level 6) mountain range, or lower when level 6 is missing). In such cases, the grouped features were assigned a common GID. By contrast, if polygons from the same source did not share a single common GMBA v2.0 unit at the highest available level, they were retained as separate entries. This procedure is exemplified in Fig. 3.

The paleoglacier groups attribute tables were populated with values synthesized both in the *Metadata* and *Paleoglacier/paleoclimate Information Spreadsheets*, using attribute rules in ESRI ArcGIS Pro 3.2.0. The rules were responsible for automatically retrieving values from the spreadsheets into the feature polygons of the paleoglacier groups by matching unique identifiers (MID, PID, and GID).

For the metadata-related attributes, values were directly transferred by matching MIDs. However, as multiple PIDs may coexist in a paleoglacier group (i.e., polygon features with a common MID and GID), further harmonization was sometimes required. When a paleoglacier group contained a single PID (i.e., the same values for chronology, and derived paleoclimate attributes), values were directly transferred. In contrast, for paleoglacier groups with multiple PIDs (i.e., different values for chronology and/or derived paleoclimate attributes), we used ArcGIS Arcade expressions to calculate summary statistics (maximum, minimum, mean values, and range-derived uncertainty). This approach harmonized the visualization of attributes values across paleoglacier groups, while preserving the traceability of individual input values in the accompanying spreadsheets.

The harmonization of paleoglacier/paleoclimate values for attribute visualization involved two steps: (i) converting all values into common units (ages in years BP, ELAs in meters above sea level, temperatures in °C relative to present), and (ii) deriving a single representative value per paleoglacier group using summary statistics (mean, maximum, minimum, or range-derived uncertainty) previously calculated from the reported values. Importantly, we did not re-estimate any values by standardizing the methodology for derived outputs nor recalibrated chronological ages; instead, all published estimates were preserved verbatim, thereby retaining the original uncertainty of the sources. Table 1 summarizes the harmonization methodology used to derive a single representative value per paleoglacier group.

**Table 1:** Standardization methodology applied to paleoglacier and paleoclimate attributes in GLACIMONTIS.

Attribute field (Alias)	Standardization methodology
Start of Deglaciation Lower Limit (yr BP)	For each paleoglacier group, which can contain several reported ages, we retrieved the maximum value as the representative timing for the whole group.
Start of Deglaciation Lower Limit Uncertainty (yr)	Corresponds to the uncertainty attached to the retrieved start of deglaciation lower limit value selected as representative for the paleoglacier group.
Start of Deglaciation Upper Limit (yr BP)	For each paleoglacier group, which can contain several reported ages, we retrieved the minimum value as the representative timing for the whole group.
Start of Deglaciation Upper Limit Uncertainty (yr)	Corresponds to the uncertainty attached to the retrieved start of deglaciation upper limit value selected as representative for the paleoglacier group.
Modern Equilibrium Line Altitude (m)	For each paleoglacier group, we calculated the average of all modern ELA reported across its PIDs.
Paleo Equilibrium Line Altitude (m)	For each paleoglacier group, we calculated the average of all paleo ELA estimated across its PIDs.
Paleo Equilibrium Line Altitude Uncertainty (m)	For each paleoglacier group, we calculated the uncertainty as the range required to encompass the minimum and maximum reported paleo ELA values across all PIDs. This was expressed as a symmetric error margin (±) around the mean paleo ELA.
Delta Equilibrium Line Altitude - Minimum (m)	For each paleoglacier group, we retrieved (if given in the original source) or calculated (if absent in the original source) the minimum reported difference between modern and paleo ELA values across its PIDs.
Delta Equilibrium Line Altitude - Maximum (m)	For each paleoglacier group, we retrieved (if given in the original source) or calculated (if absent in the original source) the maximum reported difference between modern and paleo ELA values across its PIDs.
Temperature Depression Minimum (°C)	For each paleoglacier group, we retrieved the minimum reported estimate of temperature depression relative to present across its PIDs.
Temperature Depression Maximum (°C)	For each paleoglacier group, we retrieved the maximum reported estimate of temperature depression relative to present across its PIDs.

Although this harmonization enables consistent comparisons across mountain ranges and supports broad-scale analyses, it inevitably reduces spatial variability and obscures some local detail. Some publications report multiple ELAs for individual valleys or for different aspects of an ice cap, while others provide only a single regional ELA. In our framework, such information is summarized at the paleoglacier group scale. As a result, the dataset captures robust regional patterns but does not retain all glacier-specific dynamics. Individual input values of chronology and paleoclimate patterns can still be checked in the *Paleoglacier/Paleoclimate Information Spreadsheet*. However, if users require detailed values of chronology and paleoclimate at individual paleoglacier scale, they should refer directly to the source publications for full local context.

### Filtered mountain paleoglacier extents

The *Filtered Mountain Paleoglacier Extents* feature dataset provides a simplified and uniform layer representing the most likely estimation of the maximum glacier areal extent during the LLGM. It contains a single feature class, *Filtered Reconstructed Paleoglaciers*, which is derived from the *Empirically Reconstructed Paleoglaciers* feature class (see *Mountain Paleoglacier Extents*).

Unlike the *Mountain Paleoglacier Extents* feature dataset, which preserves all published reconstructions side by side without modifying their outlines, the *Filtered Mountain Paleoglacier Extents* feature dataset is a curated output where we selectively included and excluded reconstructions or parts of a reconstruction from different sources. It represents a generalized output representative of the maximum glacier extent during the LLGM. In doing so, this dataset does not include overlapping reconstructions, does not retain identifiers (MID, PID, GID), is not hierarchically grouped by the GMBA v2.0^90,91^, while retaining the multi-resolution aspect of original sources. Although direct traceability is not possible due to the loss of identifiers, it is still possible to indirectly trace the polygon features of the *Filtered Mountain Paleoglaciers* feature class by overlaying with the *Empirical Reconstructed Paleoglaciers* feature class.

Using ESRI ArcGIS Pro 3.2.0^82^, we systematically identified and manually edited regions where multiple glacier reconstructions overlapped. First, overlapping reconstructions were flagged by applying topology rules within the *Empirically Reconstructed Paleoglaciers* feature class, which flagged polygons that intersected within the feature class. Second, reconstructions that were incomplete (e.g., truncated by political boundaries or restricted to partial study areas) or judged less plausible based on spatial coherence with surrounding evidence (e.g., discontinuous margins, isolated fragments inconsistent with mapped landforms) were filtered out. Third, where two or more reconstructions overlapped substantially and were considered equally plausible, we selectively combined their extents into a single outline using manual editing tools (e.g., edit, union, and erase operations). Fourth, in cases where reconstructions contained both plausible and implausible sections, polygons were selectively retained, with problematic segments removed.

Consequently, the dataset is not suitable for analyses of individual reconstructions outlines or detailed regional glaciation patterns, due to its modifications and lack of direct reference to original publications. Also, we do not recommend using this dataset for constraining chronological ages, as it is representative of the possible last maximum extent for a given region. For such applications, users should refer to the *Empirically Reconstructed Paleoglaciers* feature class. Instead, it is intended as a ready-to-use glacier mask for inter- and transdisciplinary studies, such as for climate, cryosphere, and ecological modeling, particularly at coarser spatial resolutions and broad-scale applications.

### Glaciated mountain ranges overview

The *Glaciated Mountain Ranges Overview* feature dataset summarizes the extent to which mountain ranges were glaciated during the LLGM. It contains a single feature class, *Glaciated Mountain Ranges*. The dataset provides a global overview of where glaciation was documented, absent, or uncertain, thereby highlighting both data gaps and the current state of knowledge. Each mountain range (GMBA v2.0, Level 3)^90,91^ was assigned two attributes:

1. Glaciation status – classified as (i) glaciated (yes), (ii) unglaciated (no), or (iii) unassessed (unassessed) during the LLGM.
2. Reconstruction availability: classified as available (yes) or not available (no).

In addition to categorical attributes, an *Additional comments* field was included for each mountain range. This field provides concise qualitative notes that capture context not fully represented by the classification. Examples include: (i) geographical variation within a range (e.g., one sector glaciated while another remained ice-free), (ii) uncertainty about the completeness or quality of available reconstructions (e.g., schematic or generalized outlines), (iii) geomorphological evidence suggesting possible but unconfirmed glaciation (e.g., cirque forms without mapped reconstructions), and (iv) explanations for unglaciated classifications (e.g., insufficient elevation relative to regional paleo ELA). These comments serve to highlight heterogeneity, ambiguity, or research gaps that may be important for regional or local analyses, while categorical classification provides a standardized framework for global synthesis.

The classification was based primarily on the GLACIMONTIS compilation of reconstructions, supplemented with regionally reported paleo ELA estimates, published geomorphological evidence, and, where necessary, expert interpretation. Classification rules for both glaciation status and reconstruction availability are summarized in Table 2.

**Table 2:** Classification rules for glaciation status and reconstruction availability in GLACIMONTIS.

Glaciation status		Reconstruction availability	
Category	Decision rule	Reconstruction available	Reconstruction not available
Glaciated (during the LLGM)	The mountain range exhibits evidence of glaciation during 57-14 kyr BP, supported by corroborative glacier reconstruction, geomorphological and/or chronological research	One or more glacier reconstruction(s) available in the GLACIMONTIS	No glacier reconstruction is currently available in GLACIMONTIS; however, independent geomorphological and chronological studies provide robust evidence of former glaciation
Unassessed	The existing evidence of glaciation is too weak or conflicting to confirm the presence or absence of glaciation.	Condition is not applicable. Absence of reconstructions is required	This category also includes areas where summits could have supported glaciation but have not yet been systematically investigated, as well as regions with sparse or contradictory literature, where some studies suggest glaciation while others reject it
Unglaciated	Studies explicitly indicate absence of glaciers during the LLGM; or maximum elevations consistently below reported paleo ELA values	Condition is not applicable. Absence of reconstruction is required	Literature consensus that the range was ice-free; summits below paleo ELA threshold

All classifications were applied at GMBA^90,91^ Level 3 (e.g., Central European Highlands, Eastern Rift Mountains). This level provides an optimal balance between detail, interpretability, and global consistency. Coarser levels (1–2) are too broad to capture regional variation in glaciation. Finer levels (4–6) are inconsistent across regions, and often divide ranges into small subunits for which reconstructions or geomorphological evidence are not consistently available. For example, in South America the entire Cordillera Oriental from Ecuador to Chile is represented as a single Level 4 unit, whereas in France Level 4 subdivides the country into five separate ranges. In addition, in many regions higher levels of subdivision do not exist (e.g., some ranges terminate at Level 4, with no corresponding Level 5). Therefore, level 3 offers an appropriate scale for sub-continental to regional scale assessment while acknowledging that smaller subranges within broader units are not totally resolved.

## Data Records

The GLACIMONTIS geodatabase is archived in *Zenodo* and is available at 10.5281/zenodo.15600659. The dataset is openly accessible under a CC-BY- 4.0 license. The geodatabase includes two spreadsheets, four main feature datasets, and five additional base layers. Here we detail their content and structure.

### GLACIMONTIS worksheet

The *GLACIMONTIS Worksheet* consists of two spreadsheets: the *Metadata Information Spreadsheet* and the *Paleoglacier/paleoclimate Information Spreadsheet*, which collectively provides an overview of the literature, paleoglacier reconstructions and their associated metadata. It is presented as an excel file (*.xlsx*) and as two standalone tables in the GLACIMONTIS geodatabase (*.dbf*).

### Metadata information spreadsheet

Bibliographic and methodological metadata for each source publication, including reference details, data availability, and chronology methods (attributes defined in Table 3). This spreadsheet is organized by MID and available within the *GLACIMONTIS worksheet.xlsx* or within the *GLACIMONTIS.gdb*.

**Table 3:** Metadata Information Spreadsheet: Overview of the metadata fields collected for each publication in the literature review.

Field	Alias	Description	Data Type	Category
mid	Metadata Identifier (MID)	Publication unique identifier related to metadata information	Text	Identification
continent	Continent	Continent where the study site(s) of the publication are located	Text	Spatial
reference	Reference	Full reference of the publication	Text	Referencing
doi	DOI Code	Digital object identifier of the publication	Text	Referencing
publititle	Publication Title	Publication title with direct link to original publication	Text	Referencing
scitation	Short Citation	Short citation of the publication	Text	Referencing
yrpubli	Year of Publication	Year the publication or database was published	Numeric	Referencing
addrefs	Additional References	Additional references in the original publication for the glacier reconstructions	Text	Referencing
dataacq	Data Availability	Indicates how the glacier reconstruction was retrieved from the original publication	Text	Referencing
datasource	Data Availability Source	Indicates the source where reconstructions were retrieved from if open access	Text	Referencing
geomap	Geomorphological Mapping	Indicates if a geomorphological map is provided by the referenced publication, where 1 = True and 0 = False	Numeric	Methodology
quantchrono	Quantitative Chronology	Indicated if any quantitative chronology method were used by referenced study/publication, where 1 = True and 0 = False	Numeric	Methodology
cosmodating	Cosmogenic Nuclide Dating	Indicates if cosmogenic nuclide dating methods were used by the referenced study/publication, where 1 = True and 0 = False	Numeric	Methodology
addchrono	Additional Chronology Methods	Indicates if other dating methods, where used where 1 = True and 0 = False	Numeric	Methodology
adddating	Additional Chronology Methods Applied	Indicates which additional dating methods were applied, if applicable	Text	Methodology

The spreadsheet includes the following columns: Field (attribute name), Alias (brief explanation or identifier for the field), Description (details about the field’s purpose or content), Data Type (type of data stored in the field), and Category (thematic grouping, e.g., Identification, Referencing, Methodology).

### Paleoglacier/paleoclimate information spreadsheet

Selected quantitative findings from the source publications, including chronology, modern and paleo ELAs, and temperature cooling estimates (attributes defined in Table 4). This spreadsheet is organized by MID and PID, and available in the *GLACIMONTIS worksheet.xlsx* and within the *GLACIMONTIS.gdb*.

**Table 4:** Paleoglacier/paleoclimate Information Spreadsheet: Overview of the attribute fields recorded for each spatial feature in the [Metadata Information Spreadsheet].

Field	Alias	Description	Data Type	Category
mid	Metadata Identifier (MID)	Publication unique identifier related to metadata information	Text	Identification
pid	Paleoglacier Identifier (PID)	Publication study site/paleoglacier identifier, related to publication findings	Text	Identification
gid	Geographic Identifier (GID)	Spatial identifier of polygon feature shapes based on mountain range intersection	Text	Identification
continent	Continent	Continent where the study site(s) of the publication are located	Text	Spatial
deglalow	Start of Deglaciation Lower Limit (yr BP)	Maximum estimated timing of the glaciation in years, i.e., oldest estimated age	Numeric	Findings
deglalowun	Start of Deglaciation Lower Limit Uncertainty (yr)	Estimated uncertainty for the start of deglaciation lower limit in years	Numeric	Findings
deglaupp	Start of Deglaciation Upper Limit (yr BP)	Minimum estimated timing of the glaciation in years, i.e., youngest estimated age	Numeric	Findings
deglauppun	Start of Deglaciation Upper Limit Uncertainty (yr)	Estimated uncertainty for the start of deglaciation upper limit in years	Numeric	Findings
modela	Modern Equilibrium Line Altitude (m)	Present-day equilibrium-line altitude in meters above present day sea level	Numeric	Findings
paleoela	Paleo Equilibrium Line Altitude (m)	Equilibrium-line altitude during the LLGM in meters above present day sea level	Numeric	Findings
paleoelaun	Paleo Equilibrium Line Altitude Uncertainty (m)	Error margin for the paleo ELA estimate in meters	Numeric	Findings
delamin	Delta Equilibrium Line Altitude - Minimum (m)	Difference (lowest value) in meters between present-day and paleo ELA	Numeric	Findings
delamax	Delta Equilibrium Line Altitude - Maximum (m)	Difference (highest value) in meters between present-day and paleo ELA	Numeric	Findings
coolmin	Temperature Depression Minimum (°C)	Temperature depression (minimum) from present-day temperature to paleo temperature in degree Celsius	Numeric	Findings
coolmax	Temperature Depression Maximum (°C)	Temperature depression (maximum) from present-day temperature to paleo temperature in degree Celsius	Numeric	Findings

The spreadsheet includes the following columns: Field (attribute name), Alias (brief explanation or identifier for the field), Description (further details about the field’s purpose or content), Data Type (format of the data, e.g., Numeric), and Category (thematic grouping, e.g., Identification for ID numbers, Findings for quantitative results).

### Feature datasets

The GLACIMONTIS geodatabase contains four primary feature datasets. The feature datasets are provided withing the GLACIMONTIS geodatabase (.gdb), and its feature classes are also provided as*.shp* and*.kml/.kmz*. In the case of the *Filtered Mountain Paleoglaciers*, a netCDF (*.nc*) file is also provided for integration with follow up glaciological models, for example.

1. *Mountain Paleoglacier Extents* – all empirically reconstructed glacier polygons, with associated constraint lines.
2. *Filtered Mountain Paleoglacier Extents* – subset of reconstructions filtered from the *Empirically Reconstructed Paleoglaciers*.
3. *Glaciated Mountain Ranges Overview* – classification of mountain ranges (GMBA v2.0^90,91^, Level 3) by status of glaciation, availability of reconstruction and additional notes about glacial research in the area.
4. *Technical Validation* – dataset used to validate the completeness, accuracy and quality of the GLACIMONTIS geodatabase.

Each feature dataset contains feature classes and attribute tables (Fig. 2). We refer to the ‘Attribute table’ as any numerical and textual tabular information when spatially connected to polygon feature shapes from a feature class.

### Mountain paleoglacier extents dataset

Spatial feature dataset containing empirically reconstructed glacier extents, along with constraint lines, organized by MID and GID (attributes defined in Supplementary Table 1). Available within the *GLACIMONTIS.gdb*, and as *EmpiricallyReconstructedPaleoglaciers.shp*, *EmpiricallyReconstructedPaleoglaciers.kml, ReconstructionConstraintLines.shp*, and *ReconstructionConstraintLines.kml*.

The geodatabase was compiled using the sources presented in the Data Supplementary Information 1 (Data S1) and in the *Zenodo* data repository (10.5281/zenodo.15600659).

### Filtered mountain paleoglacier extents dataset

Subset of empirically reconstructed glacier extents filtered for broad-scale global applications (attributes defined in Table 5). Available within the *GLACIMONTIS.gdb*, and the *FilteredReconstructedPaleoglaciers.shp*, *FilteredReconstructedPaleoglaciers.kml*, and *FilteredReconstructedPaleoglaciers.nc*.

**Table 5:** Attribute table of *Filtered Reconstructed Paleoglaciers* feature class.

Field	Alias	Description	Data Type	Category
OBJECTID	OBJECTID	ESRI ArcGIS Pro’s feature class automated object identifier	Numeric	Identification
SHAPE	Shape	ESRI ArcGIS Pro’s feature class automated shape geometry class	Text	Spatial
GlobalID	GlobalID	Automatically generated global shape identifier through the datasets	Text	Identification
area	Area (km²)	Total area in square kilometers of the polygon feature shape	Numeric	Spatial
perimeter	Perimeter (km)	Total perimeter in kilometers of the polygon feature shape	Numeric	Spatial
SHAPE_AREA	Area (°²)	ESRI ArcGIS Pro’s feature class automated geometry calculation derived from coordinate system. It represents the total area of the polygon feature shape in square degrees, a geographic unit based on latitude and longitude. Not visible for GLACIMONTIS purposes	Numeric	Spatial
SHAPE_LENGTH	Perimeter (°)	ESRI ArcGIS Pro’s feature class automated geometry calculation derived from coordinate system. It represents the total area in degrees of the polygon feature shape. Not visible for GLACIMONTIS purposes	Numeric	Spatial

### Glaciated mountain ranges overview dataset

Feature dataset summarizing the status of glaciation, availability of reconstruction and timing of glaciation for each GMBA v2.0 level 3^90,91^ mountain range (attributes defined in Table 6). Available within the *GLACIMONTIS*.gdb, and *GlaciatedMountainRangesOverview.shp* and *GlaciatedMountainRangesOverview.kml*.

**Table 6:** Attribute table of Glaciated Mountain Ranges feature class.

Field	Alias	Description	Data Type	Category
OBJECTID	OBJECTID	ESRI ArcGIS Pro’s feature class automated object identifier	Numeric	Identification
GlobalID	GlobalID	Automatically generated global shape identifier through the datasets	Text	Identification
SHAPE	Shape	ESRI ArcGIS Pro’s feature class automated shape classification	Text	Spatial
mrange3	Mountain Range Level 3	GMBA v2.0 (level 3)^90,91^ mountain ranges classification	Text	Spatial
glaciated	Glaciation Status	Indicates if the mountain range has been glaciated at the LLGM according to GLACIMONTIS	Text	Findings
reconstruct	Reconstruction Availability	Indicates if reconstructed paleoglacier(s) are available for the given mountain range	Text	Findings
comments	Additional Comments	Additional comments regarding glaciation, data and research development for a given mountain range	Text	Findings
area	Area (km²)	Total area in square kilometers of the selected mountain range	Numeric	Spatial
perimeter	Perimeter (km)	Total perimeter in kilometers of the selected mountain range	Numeric	Spatial
SHAPE_AREA	Area (°²)	ESRI ArcGIS Pro’s feature class automated geometry calculation derived from coordinate system. It represents the total area in square degrees of the polygon feature shape. Not visible for GLACIMONTIS purposes	Numeric	Spatial
SHAPE_LENGTH	Perimeter (°)	ESRI ArcGIS Pro’s feature class automated geometry calculation derived from coordinate system. It represents the total area in degrees of the polygon feature shape. Not visible for GLACIMONTIS purposes	Numeric	Spatial

### Technical validation dataset

Feature dataset documenting the completeness of the GLACIMONTIS geodatabase (attributes defined in Table 7). Accessible within the *GLACIMONTIS.gdb*, and as *TechnicalValidation.shp* and *TechnicalValidation.kml*.

**Table 7:** Attribute table of the *Technical Validation* dataset.

Field	Alias	Description	Data Type	Category
OBJECTID	OBJECTID	ESRI ArcGIS Pro’s feature class automated object identifier	Numeric	Identification
SHAPE	Shape	ESRI ArcGIS Pro’s feature class automated shape classification	Text	Spatial
mrange4	Mountain Range Level 4	GMBA v2.0 (level 4)^90,91^ mountain ranges classification	Text	Spatial
availrecons	Available Reconstruction	Technical validation classification output. It classifies mountain ranges based on the advancements provided by GLACIMONTIS	Text	Findings
area	Area (km²)	Total area in square kilometers of the selected mountain range	Numeric	Spatial
perimeter	Perimeter (km)	Total perimeter in kilometers of the selected mountain range	Numeric	Spatial
SHAPE_AREA	Area (°²)	ESRI ArcGIS Pro’s feature class automated geometry calculation derived from coordinate system. It represents the total area of the polygon feature shape in square degrees. Not visible for GLACIMONTIS purposes	Numeric	Spatial
SHAPE_LENGTH	Perimeter (°)	ESRI ArcGIS Pro’s feature class automated geometry calculation derived from coordinate system. It represents the total area of the polygon feature shape in square degrees. Not visible for GLACIMONTIS purposes	Numeric	Spatial

### Base layers

Supporting geographic and topographic reference layers (administrative boundaries, global relief model, reconstructed ice sheets, and GMBA mountain ranges) included to provide spatial context.

### World administrative boundaries

Geopolitical and administrative division of land with international recognition as country^86^ (https://public.opendatasoft.com/explore/dataset/world-administrative-boundaries/information/), presented as polygon feature shape within the *GLACIMONTIS.gdb*. The outlines and attribute table from this layer were preserved fully from original source (for further details we refer to the original source).

### Ice sheets at the LGM

The ice sheet reconstructions showing the maximum ice extent at the LGM were acquired from Batchelor *et al*.^87,88^ (https://osf.io/7jen3/overview) for the Northern Hemisphere (Eurasian, Laurentide, Cordillera, Iceland and Greenland ice sheets), Bentley *et al*.^22^ for Antarctica ice sheet, Davies *et al*.^45^ for the Southern part of the Patagonian ice sheet and Clapperton^81^ for the Northern part of the Patagonian ice sheet, presented as polygon feature shape (attributes defined in Table 8). Available within GLACIMONTIS.gdb. We did not provide them as independent layers; users can access them from the original sources.

**Table 8:** Attribute table of the ice sheets at the LGM base layer.

Field	Alias	Description	Data Type	Category
OBJECTID	OBJECTID	ESRI ArcGIS Pro’s feature class automated object identifier	Numeric	Identification
SHAPE	Shape	ESRI ArcGIS Pro’s feature class automated shape classification	Text	Spatial
icesheet	Ice Sheet	Name of the ice sheet and/or ice sheet complex	Text	Identification
reference	Reference	Full reference of the publication	Text	Referencing
doi	DOI code	Digital Object Identifier of the publication	Text	Referencing
scitation	Short Citation	Short citation of the publication	Text	Referencing
yrpubli	Year of Publication	Year the publication or database was published	Numeric	Referencing
area	Area (km²)	Total area in square kilometers of the selected mountain range	Numeric	Spatial
perimeter	Perimeter (km)	Total perimeter in kilometers of the selected mountain range	Numeric	Spatial
SHAPE_AREA	Area (°²)	ESRI ArcGIS Pro’s feature class automated geometry calculation derived from coordinate system. It represents the total area of the polygon feature shape in square degrees. Not visible for GLACIMONTIS purposes	Numeric	Spatial
SHAPE_LENGTH	Perimeter (°)	ESRI ArcGIS Pro’s feature class automated geometry calculation derived from coordinate system. It represents the total area of the polygon feature shape in square degrees. Not visible for GLACIMONTIS purposes	Numeric	Spatial

### Global relief model

NOAA ETOPO global relief model with 30 arc-sec resolution bedrock version^89^ (https://www.ncei.noaa.gov/products/etopo-global-relief-model). It integrates topography, bathymetry, and shoreline data. The top layer of present-day ice sheets covering Greenland and Antarctica are removed, but we retained the present-day mountain glacier thickness. It is available within the *GLACIMONTIS.gdb* as raster file (*.tiff*). We did not provide them as independent layers; users can access them from the original source.

### Mountain range level 1–6

Global hierarchical mountain range classification extracted from the GMBA v2.0^90,91^ (https://www.earthenv.org/mountains), attributes defined in Table 9. In the GLACIMONTIS geodatabase only mountain ranges from level 1 to level 6 are present, meanwhile in the original source mountain ranges can reach up to level 10 in selected regions. It is available as individual layers, one for each mountain range level, within the *GLACIMONTIS.gbd*. We did not provide them as independent layers; users can access them from the original source.

**Table 9:** Attribute table of the Mountain Ranges Level 1–6 base layers.

Field	Alias	Description	Data Type	Category
OBJECTID	OBJECTID	ESRI ArcGIS Pro’s feature class automated object identifier	Numeric	Identification
SHAPE	Shape	ESRI ArcGIS Pro’s feature class automated shape classification	Text	Spatial
mrange1–6	Mountain Range Level 1–6	Name of the ice sheet and/or ice sheet complex	Text	Identification
area	Area (km²)	Total area in square kilometers of the selected mountain range	Numeric	Spatial
perimeter	Perimeter (km)	Total perimeter in kilometers of the selected mountain range	Numeric	Spatial
SHAPE_AREA	Area (°²)	ESRI ArcGIS Pro’s feature class automated geometry calculation derived from coordinate system. It represents the total area of the polygon feature shape in square degrees. Not visible for GLACIMONTIS purposes	Numeric	Spatial
SHAPE_LENGTH	Perimeter (°)	ESRI ArcGIS Pro’s feature class automated geometry calculation derived from coordinate system. It represents the total area of the polygon feature shape in square degrees. Not visible for GLACIMONTIS purposes	Numeric	Spatial

## Data Overview

**Fig. 4:**
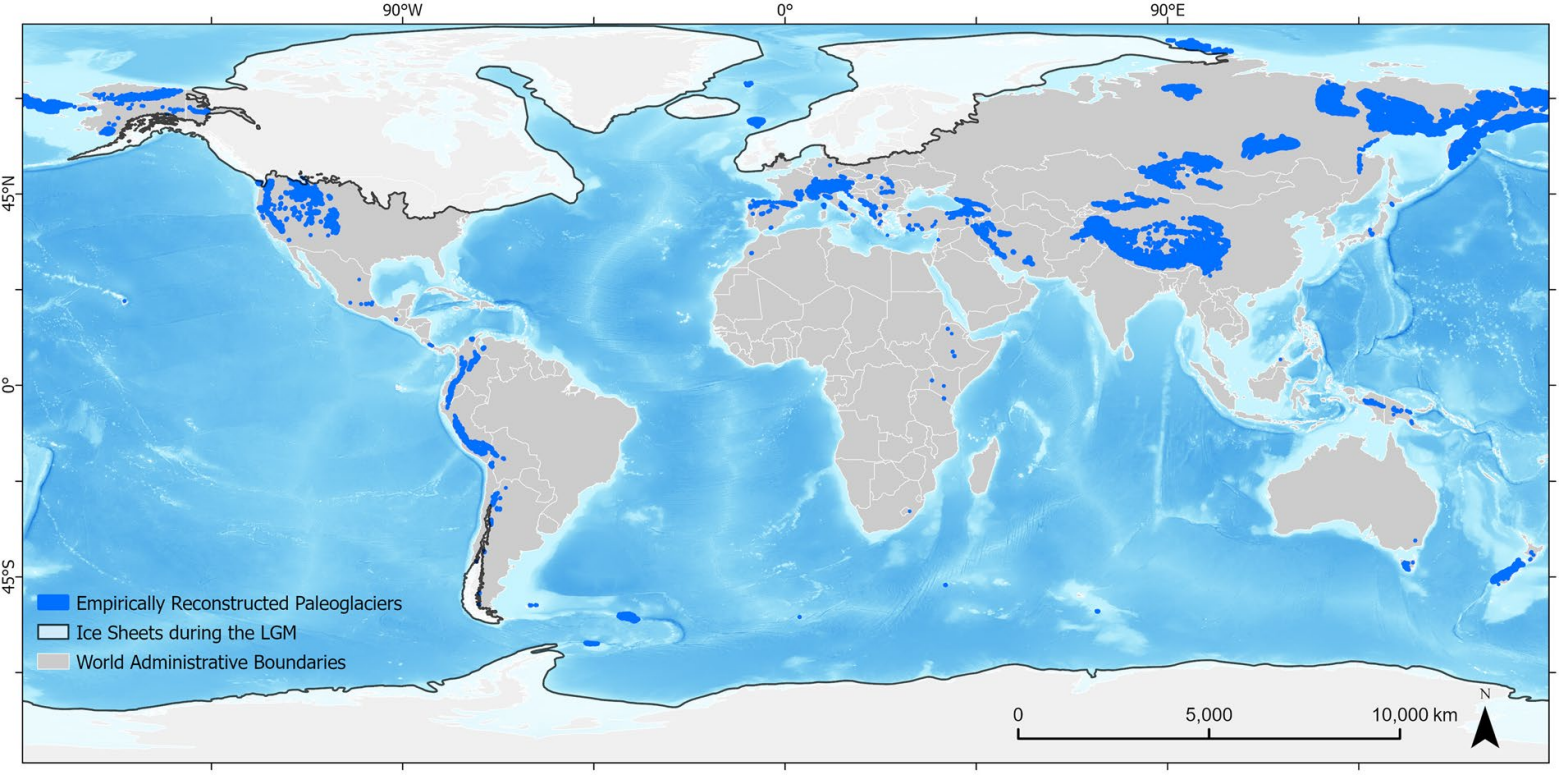
Mountain Paleoglacier Extents dataset in GLACIMONTIS. All reconstructed paleoglaciers (blue), and ice sheets (white)^22,45,81,87,88^ compiled in the GLACIMONTIS geodatabase. The paleoglacier polygon boundaries were exaggerated for visualization purposes. Background shows the base layers representing present-day global ocean relief model^89^ and present-day land delimitation with geopolitical divisions^86^.

A total of 15,014 individual paleoglacier reconstructions are compiled in the *Mountain Paleoglacier Extents feature dataset* (Fig. 4). GLACIMONTIS geodatabase features 8,809 reconstructions of paleoglaciers at the LLGM compiled for the first time, excluding ice sheets. This number was achieved by selecting out each individual polygon reconstructions in GLACIMONTIS where the reference or additional references contained any mention to Ehlers *et al*., either the 2004^36^ or 2011^37^ book.

**Fig. 5:**
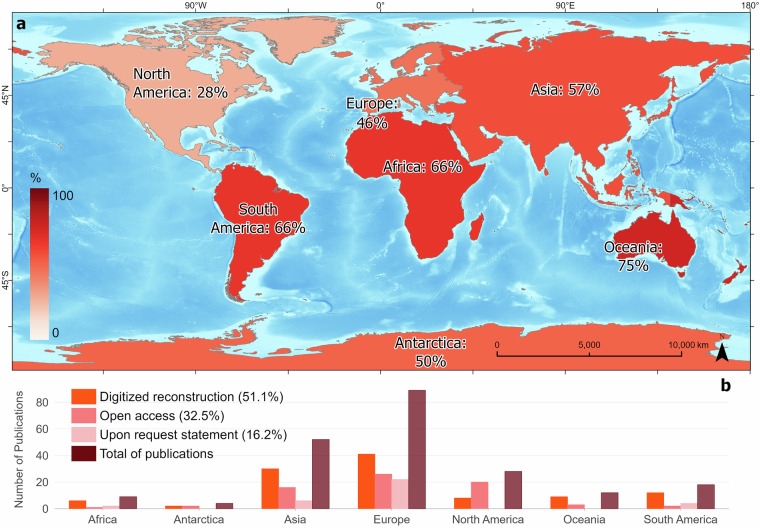
Data Acquisition Methods. (**a**) Map showing the percentage of publications per continent where glacier reconstructions were digitized from figures, relative to the total number of publications reviewed in that continent. A value of 0% indicates that all data for that continent were obtained through open-access repositories or provided upon request, while 100% indicates that all data were acquired via digitization by the authors of this publication. (**b**) Bar chart illustrating the data acquisition methods used across continents by absolute number of publications. Percentages in the legend indicate the share of each method relative to the entire literature review.

Despite this extensive coverage, more than half of the reviewed source publications required digitization and georeferencing (51.1%), since their reconstructions were not originally available in digital format. In total, they represent reconstructions from 107 sources, of which 61 sources had their reconstructions published after the year 2011. Only 32.5% were openly accessible, while a further 16.2% were available upon request. Figure 5 shows how data accessibility and publication volume varied strongly by continent, highlighting the uneven global distribution of paleoglacier reconstructions and open-access data.

**Fig. 6:**
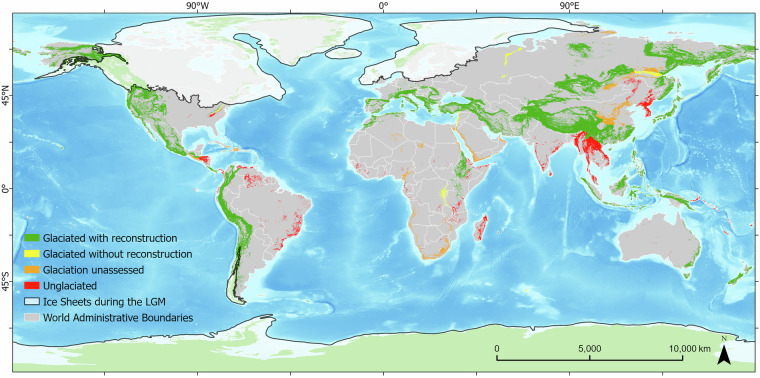
Glaciated Mountain Ranges Overview in GLACIMONTIS. Classification of mountain ranges (GMBA, level 3) regarding status of glaciation and/or glaciological research during the LLGM, where 1) glaciated with reconstruction shows mountain ranges where at least one glacier has been reconstructed for the LLGM period, 2) glaciated without reconstruction shows mountain ranges with proved glaciation at the LLGM but lacking reconstructions and paleoglacier dimensions, 3) glaciation unassessed shows mountain ranges where geomorphological evidence is not sufficient to stablish the presence or absence of glaciers during the LLGM, and 4) Unglaciated shows mountain ranges with unlikely presence of paleoglaciers or proved to be glacier free during our time frame. Base map shows present-day global ocean relief model^89^, present-day land delimitation^86^, and ice sheet outlines during the LGM^22,45,81,87,88^.

Beyond quantifying the total number of reconstructions and their accessibility (Figs. 4, 5), we also created a mountain-range classification of glaciation. In total, 177 mountain ranges were classified according to their glaciation history: 94 were glaciated during the LLGM (84 with reconstruction(s) and 10 without, but were interpreted by the authors to have been partially glaciated at the LLGM), 67 were unglaciated, and 16 remain unassessed due to insufficient geomorphological evidence. Figure 6 shows the global distribution of these classifications.

## Technical Validation

The *Technical Validation* feature dataset provides a quantitative comparison of the spatial coverage between the *Mountain Paleoglacier Extents* feature dataset in the GLACIMONTIS geodatabase and the reconstructions documented in the Ehlers *et al*. (2011) geodatabase^37^, excluding mountain ranges that were totally covered by ice sheets at the LGM. We based our analysis using the GMBA v2.0^90,91^ level 4 mountain ranges, which provide the highest level of detail that is consistently defined worldwide. In many regions, higher levels of subdivision (Level 5 and above) are not available.

**Fig. 7:**
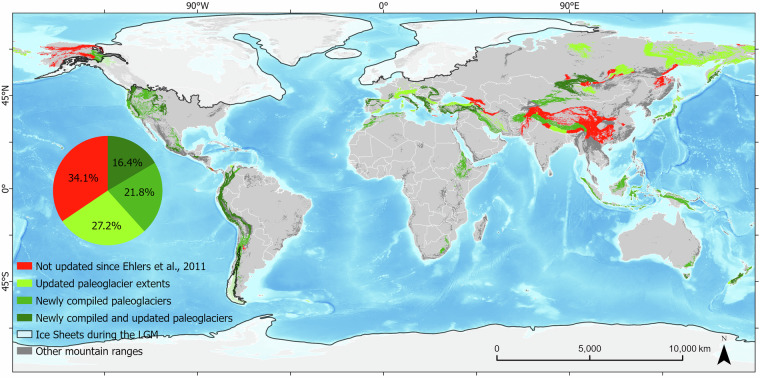
Global distribution of mountain ranges categorized by reconstruction status in GLACIMONTIS. Mountain ranges are color-coded based on their classification. The map highlights the regions where GLACIMONTIS has expanded or refined existing paleoglacier data in comparison to Ehlers *et al*.^37^. Base layer: GMBA v2.0^90,91^, global relief model^89^, World administrative boundaries^86^ and ice sheet outlines during the LGM^22,45,81,87,88^.

We analyzed 208 mountain ranges, of which 135 ranges were updated with new or revised LLGM glacier reconstructions in GLACIMONTIS, while 71 ranges retained their coverage from the Ehlers *et al*. geodatabase^37^ without updates (Fig. 7). The classification was performed in ESRI ArcGIS Pro 3.2.0^82^ into four categories:

1. No update since Ehlers *et al*. (2011): Mountain ranges where no new reconstructions or updates to the paleoglacier extents have been made, maintaining the same coverage as documented in Ehlers *et al*.^37^.
2. Updated paleoglacier extents: Mountain ranges where reconstructions were previously available in Ehlers *et al*.^37^ but where new or additional reconstructions now exist. Thus, the reconstructions presented in GLACIMONTIS only update and/or provide a new interpretation of the paleoglacier extent in these regions (e.g., by modification to the glacier outlines).
3. Newly compiled paleoglaciers: Mountain ranges where GLACIMONTIS includes glacier reconstructions in locations where reconstructions were not available in Ehlers *et al*.^37^.
4. Updated and newly compiled paleoglaciers: Mountain ranges where both updated (category 2) and newly compiled reconstructions (category 3) now exist in comparison to Ehlers *et al*.^37^.

The above categories were determined as follows: A GLACIMONTIS subset was created for validation by removing reconstructions that had already been documented in Ehlers and Gibbard^36^ and Ehlers *et al*.^37^. Mountain ranges that intersected with the Ehlers *et al*.^37^ geodatabase were then identified and compared against those intersecting the subset GLACIMONTIS dataset. Subtracting the two selections allowed the identification of mountain ranges containing only reconstructions from Ehlers *et al*.^37^. These ranges were classified as not having been updated (category 1). To identify updated glacier outlines, polygons from the subset GLACIMONTIS dataset were selected based on their intersection with Ehlers *et al*.^37^, and the corresponding mountain ranges were classified accordingly (category 2). Similarly, mountain ranges intersected by polygons in the subset GLACIMONTIS dataset but not in Ehlers *et al*.^37^ were classified as having new reconstructions (category 3). Whenever a single mountain range contained both updated outlines and new reconstructions, these classifications were adjusted to reflect the combined category 4. The validation does not evaluate the completeness of glaciation knowledge for each mountain range nor does it imply that all existing glaciers during the LLGM have been reconstructed. Rather it assesses whether GLACIMONTIS provides updates (e.g., new reconstructions or reinterpretations) or retains coverage without updates since Ehlers *et al*.^37^, the last major global synthesis of glacier extents at the LLGM. Thus, missing reconstructions may occur across all categories. For individual assessments of the broader state of knowledge on glaciation for individual mountain ranges or the likely maximum-suggested ice extent from compiled reconstructions, see *Glaciated Mountain Ranges Overview* and *Mountain Paleoglacier Extents* feature dataset, respectively. We also encourage users to refer to original publications for detailed insight into LLGM glacier coverage for specific regions.

The comparison between GLACIMONTIS and the Ehlers *et al*.^37^ database demonstrates significant advancements in the documentation of the last maximum glacier areal extents at LLGM, particularly in regions that were previously underrepresented or lacked detailed reconstructions. By incorporating updated and newly reconstructed paleoglaciers, GLACIMONTIS enhances the spatial and temporal resolution of paleoglacier datasets, addressing critical gaps in global coverage. This expansion not only reflects the progress made in paleoglaciology over the past decade but also underscores the dynamic nature of research in this field. While 71 mountain ranges remain with no new reconstruction since Ehlers *et al*.^37^, the inclusion of metadata and citation details in GLACIMONTIS enables future researchers to re-investigate these regions. Overall, the *Technical Validation* highlights the value of GLACIMONTIS as a robust and versatile resource for studies across multi-disciplinary fields within Quaternary science, offering a foundation for improved modeling of past climates, glacier dynamics, and ecosystem interactions during the LLGM.

## Usage Notes

As previously addressed by earlier global compilations^36,37,44^, GLACIMONTIS is a large, evolving dataset and may contain errors or omissions. User feedback reporting mistakes or providing improved outlines, chronologies, or metadata are welcome and can be shared with the corresponding authors at mountain.paleoglaciers@uib.no. Verified corrections will be incorporated in future releases with proper attribution, and version changes will be documented in the changelog.

We kindly ask users to cite not only this geodatabase but also the original sources when using or referring to specific, individual reconstructions contained within it. These reconstructions were originally published in independent studies and were generously shared with us by their authors in the spirit of open science. Proper attribution ensures that the original contributors receive due credit for their work and helps sustain a culture of transparency, collaboration, and continued data sharing within the scientific community.

### Literature review limitations

i) **Publication bias** – regions with long research traditions (e.g., North America, Europe) are heavily represented, while remote or politically restricted areas remain under-studied.
ii) **Language bias** – although non-English works were included if accessible, the search was primarily English-language, meaning reconstructions published only in local outlets may have been missed.
iii) **Uneven reporting** – not all studies provide complete paleoglacier/paleoclimate information (e.g., chronology, ELA, climate estimates), limiting consistency in what could be compiled.
iv) **Temporal coverage** – the literature is dynamic; new reconstructions and dating studies continue to appear, and GLACIMONTIS reflects the state of knowledge up to July 2024.
v) **Accessibility of data** – over half of reconstructions were available only through digitization of published figures, highlighting uneven data sharing practices.

### Temporal limitations

GLACIMONTIS includes reconstructions of maximum ice areal extent during the predefined time period, but several caveats apply when using the data:

i) **Undated reconstructions** (*Mountain Paleoglacier Extents & Glaciated Mountain Ranges Overview feature datasets*) – outlines without quantitative dating were mapped. In these cases, authors typically assigned them to the LGM, LLGM, or a generalized “maximum extent” during the Late Pleistocene. Such reconstructions were retained in GLACIMONTIS, as the maximum ice extent during the Late Pleistocene most often corresponds to the LGM or LLGM, but should still be treated cautiously. The attribute table explicitly indicates whether quantitative chronology was applied, allowing users to identify which reconstructions are chronologically constrained and which are not.
ii) **Dynamic glacier history** (*Mountain Paleoglacier Extents & Filtered Mountain paleoglacier Extents feature dataset)*– glaciation is inherently time-variable. GLACIMONTIS captures steady-state maximum extent only during the time interval (57-14 ka); deglacial and readvance stages are not represented, both for mountain glaciers and ice sheets.
iii) **Dating uncertainty** (*Mountain Paleoglacier Extents feature dataset*) – even when quantitative methods are applied, calibration updates and methodological differences introduce uncertainty. Reconstructions from a single location may therefore report inconsistent ages across studies. Chronological information in GLACIMONTIS is transcribed directly from the original sources and has not been recalibrated or re-estimated.
iv) **Regional asynchrony** *(Glaciated Mountain Ranges Overview feature dataset*) - labels of LLGM in GLACIMONTIS represent the asynchronous glacier maxima for different regions and glaciers worldwide. Glacier advances, retreats and peaks occurred in different timespans even within the global LGM. Users should therefore treat the GLACIMONTIS classification as a consistent framework for the last maximum ice extent within 57-14 ky BP, and consult the original publications for local details on glaciation timing.
v) **Maximum ice extent outside MIS 4/3-2/1** *(Mountain Paleoglacier Extents & Filtered Mountain Paleoglacier Extents feature dataset*) – in some regions, the maximum glacier extent occurred outside the selected interval and was excluded. For example, in Mount Chelmos (Southeast European Highlands, Hellenides, Pindus Mountains)^100^ and the Bayan Har Shan (Central Asia, Tibetan Plateau)^101^, the maximum extent predated MIS 4/3–2/1. However, because the same authors also reconstructed the maximum glacier extents within MIS 4/3–2/1, those were included in GLACIMONTIS.
vi) **Future updates** (*all products*) – some reconstructions may have had their dating revised based on new results. We encourage users needing precise temporal constraints to cross-reference landform-based chronologies (e.g., ICE-D cosmogenic nuclide database^65^).

### Spatial limitations

GLACIMONTIS compiles maximum ice areal extent reconstructions from diverse sources, but several spatial caveats apply:

i) **Uneven global coverage** (*all products*) – the density of reconstructions varies strongly by region. Europe and North America are comparatively well mapped, while Africa, parts of Asia, South America and Oceania remain underrepresented. This reflects both research effort and data accessibility.
ii) **Multi-resolution reconstructions** (*Mountain Paleoglacier Extents feature dataset*) – paleoglacier outlines are preserved in their original resolution and spatial extent. As a result, reconstructions from different sources may vary in detail, scale, and boundary definition. Local and regional comparisons should therefore be made with caution, as small glaciers in one area may be mapped in detail, while only generalized ice caps are available elsewhere.
iii) **Overlapping reconstructions** *(Mountain Paleoglacier Extents feature dataset)* – multiple studies may provide independent outlines for the same mountain area. GLACIMONTIS retains these side by side to represent interpretative diversity, rather than merging them into a single outline.
iv) **Aggregation and representativeness** (*Mountain Paleoglacier Extents feature dataset*) – for each glacier group (GID), attributes such as ELAs, temperature depressions, and deglaciation ages were aggregated across multiple reconstructions (PIDs) within that group. Standardization rules (Table 3) define how minimum, maximum, mean, or range-based values were selected. These procedures preserve comparability at the group level but inevitably reduce local detail: individual valley- or cirque-scale variations are not fully represented. Users interested in site-specific reconstructions should consult the original publications and, where available, the individual PID-level records in the GLACIMONTIS worksheet/standalone tables.
v) **Scale of GMBA framework** (*Glaciated Mountain Ranges Overview & Technical Validation*) – classification is based on GMBA v2.0 mountain ranges, which ensures global consistency but introduces mismatches in spatial resolution. For example, France is subdivided into multiple Level 4 ranges, while the entire Cordillera Oriental from Ecuador to Chile is treated as a single range. In some areas, higher levels (Level 5+) are not defined, limiting hierarchical resolution.
vi) **Georeferencing and digitization** (*Mountain Paleoglacier Extents & Filtered Mountain Paleoglacier Extents feature datasets*) – many reconstructions were digitized from published figures. This process introduces unquantified spatial uncertainties related to map scale, projection, and figure quality. Users should be cautious when applying the dataset at fine spatial scales.
vii) **Filtering and merging** (*Filtered Mountain Paleoglacier Extents feature dataset*) – the dataset provides a curated layer of glacier extents where overlapping reconstructions were combined, incomplete or implausible outlines were filtered out, and selective edits were applied (see Methods). While this produces a simplified, ready-to-use product, local details and the ability to trace polygons directly to individual sources are lost. The dataset is therefore suited only for broad-scale applications at medium to low spatial resolution. Users seeking source-specific or original resolution information should use the Empirical Paleoglacier Extents dataset instead.
viii) **Excluded areas** (*all products*) – mountain sectors covered by continental ice sheets during MIS 2 (e.g., parts of Fennoscandia, North America, Patagonia) were excluded, and therefore, glaciers that formed in mountainous regions previously covered by the ice sheets are not included (either before the ice sheet formed or once the area deglaciated).

## Supplementary information

Supplementary Table 1

Data Supplementary Information 1 (Data S1)

## Data Availability

GLACIMONTIS geodatabase, along with the reference list of all the reconstructed paleoglacier data used to compose it, can be found at 10.5281/zenodo.15600659^102^.

## Code availability

No custom scripts were used in this study. All data processing was carried out using ESRI ArcGIS Pro 3.2.0. Geoprocessing tools and workflows used are described in the Methods section.

## References

[1] Hays, J. D., Imbrie, J. & Shackleton, N. J. Variations in the Earth’s Orbit: Pacemaker of the Ice Ages: For 500,000 years, major climatic changes have followed variations in obliquity and precession. *Science* **194**, 1121–1132, 10.1126/science.194.4270.1121 (1976). 17790893 10.1126/science.194.4270.1121

[2] Schlüchter, C. The Swiss glacial record – a schematic summary. in *Developments in Quaternary Sciences* **2** 413–418, 10.1016/S1571-0866(04)80092-7 (Elsevier, 2004).

[3] Owen, L. A. & Dortch, J. M. Nature and timing of Quaternary glaciation in the Himalayan–Tibetan orogen. *Quaternary Science Reviews* **88**, 14–54, 10.1016/j.quascirev.2013.11.016 (2014).

[4] Molnar, P. & England, P. Late Cenozoic uplift of mountain ranges and global climate change: chicken or egg? *Nature* **346**, 29–34, 10.1038/346029a0 (1990).

[5] Hallet, B., Hunter, L. & Bogen, J. Rates of erosion and sediment evacuation by glaciers: A review of field data and their implications. *Global and Planetary Change* **12**, 213–235, 10.1016/0921-8181(95)00021-6 (1996).

[6] Brozović, N., Burbank, D. W. & Meigs, A. J. Climatic Limits on Landscape Development in the Northwestern Himalaya. *Science* **276**, 571–574, 10.1126/science.276.5312.571 (1997). 9110972 10.1126/science.276.5312.571

[7] Mitchell, S. G. & Montgomery, D. R. Influence of a glacial buzzsaw on the height and morphology of the Cascade Range in central Washington State, USA. *Quaternary Research* **65**, 96–107, 10.1016/j.yqres.2005.08.018 (2006).

[8] Egholm, D. L., Nielsen, S. B., Pedersen, V. K. & Lesemann, J.-E. Glacial effects limiting mountain height. *Nature* **460**, 884–887, 10.1038/nature08263 (2009). 19675651 10.1038/nature08263

[9] Herman, F. *et al*. Erosion by an Alpine glacier. *Science* **350**, 193–195, 10.1126/science.aab2386 (2015). 26450208 10.1126/science.aab2386

[10] Herman, F. *et al*. Worldwide acceleration of mountain erosion under a cooling climate. *Nature* **504**, 423–426, 10.1038/nature12877 (2013). 24352288 10.1038/nature12877

[11] Hall, A. M. & Kleman, J. Glacial and periglacial buzzsaws: fitting mechanisms to metaphors. *Quaternary Research* **81**, 189–192, 10.1016/j.yqres.2013.10.007 (2014).

[12] Hoorn, C. *et al*. The Amazon at sea: Onset and stages of the Amazon River from a marine record, with special reference to Neogene plant turnover in the drainage basin. *Global and Planetary Change* **153**, 51–65, 10.1016/j.gloplacha.2017.02.005 (2017).

[13] Flantua, S. G. A., O’Dea, A., Onstein, R. E., Giraldo, C. & Hooghiemstra, H. The flickering connectivity system of the north Andean páramos. *Journal of Biogeography* **46**, 1808–1825, 10.1111/jbi.13607 (2019).

[14] Shaw, T. E., Buri, P., McCarthy, M., Miles, E. S. & Pellicciotti, F. Local Controls on Near‐Surface Glacier Cooling Under Warm Atmospheric Conditions. *Journal of Geophysical Research: Atmospheres* **129**, e2023JD040214, 10.1029/2023JD040214 (2024).

[15] Salerno, F. *et al*. Local cooling and drying induced by Himalayan glaciers under global warming. *Nature Geoscience* **16**, 1120–1127, 10.1038/s41561-023-01331-y (2023).

[16] Gillespie, A. & Molnar, P. Asynchronous maximum advances of mountain and continental glaciers. *Reviews of Geophysics* **33**, 311–364, 10.1029/95RG00995 (1995).

[17] Petherick, L. M. *et al*. An extended last glacial maximum in the Southern Hemisphere: A contribution to the SHeMax project. *Earth-Science Reviews* **231**, 104090, 10.1016/j.earscirev.2022.104090 (2022).

[18] Clark, P. U. *et al*. The Last Glacial Maximum. *Science* **325**, 710–714, 10.1126/science.1172873 (2009). 19661421 10.1126/science.1172873

[19] Tierney, J. E. *et al*. Glacial cooling and climate sensitivity revisited. *Nature* **584**, 569–573, 10.1038/s41586-020-2617-x (2020). 32848226 10.1038/s41586-020-2617-x

[20] Annan, J. D., Hargreaves, J. C. & Mauritsen, T. A new global surface temperature reconstruction for the Last Glacial Maximum. *Climate of the Past* **18**, 1883–1896, 10.5194/cp-18-1883-2022 (2022).

[21] Seltzer, A. M., Blard, P.-H., Sherwood, S. C. & Kageyama, M. Terrestrial amplification of past, present, and future climate change. *Sci. Adv.* **9**, eadf8119, 10.1126/sciadv.adf8119 (2023). 36753551 10.1126/sciadv.adf8119 PMC9908018

[22] Bentley, M. J. *et al*. A community-based geological reconstruction of Antarctic Ice Sheet deglaciation since the Last Glacial Maximum. *Quaternary Science Reviews* **100**, 1–9, 10.1016/j.quascirev.2014.06.025 (2014).

[23] Leger, T. P. M. *et al*. A Greenland-wide empirical reconstruction of paleo ice sheet retreat informed by ice extent markers: PaleoGrIS version 1.0. *Climate of the Past* **20**, 701–755, 10.5194/cp-20-701-2024 (2024).

[24] Hughes, A. L. C., Gyllencreutz, R., Lohne, Ø. S., Mangerud, J. & Svendsen, J. I. The last Eurasian ice sheets – a chronological database and time‐slice reconstruction, DATED‐1. *Boreas* **45**, 1–45, 10.1111/bor.12142 (2016).

[25] Mark, B. G. *et al*. Tropical snowline changes at the last glacial maximum: a global assessment. *Quaternary International* **138**, 168–201, 10.1016/j.quaint.2005.02.012 (2005).

[26] Prentice, M. L., Hope, G. S., Maryunani, K. & Peterson, J. A. An evaluation of snowline data across New Guinea during the last major glaciation, and area-based glacier snowlines in the Mt. Jaya region of Papua, Indonesia, during the Last Glacial Maximum. *Quaternary International* **138–139**, 93–117, 10.1016/j.quaint.2005.02.008 (2005).

[27] Smith, J. A., Mark, B. G. & Rodbell, D. T. The timing and magnitude of mountain glaciation in the tropical Andes. *Journal of Quaternary Science* **23**, 609–634, 10.1002/jqs.1224 (2008).

[28] Mark, B. G. & Osmaston, H. A. Quaternary glaciation in Africa: key chronologies and climatic implications. *Journal of Quaternary Science* **23**, 589–608, 10.1002/jqs.1222 (2008).

[29] Hughes, P. D. & Woodward, J. C. Quaternary glaciation in the Mediterranean mountains: a new synthesis. *Geological Society, London, Special Publications* **433**, 1–23, 10.1144/SP433.14 (2017).

[30] Angel, I., Guzman, O. & Carcaillet, J. Pleistocene Glaciations in the Northern Tropical Andes, South America (Venezuela, Colombia and Ecuador). *Cuadernos de Investigación Geográfica* **43**, 571–590, 10.18172/cig.3202 (2017).

[31] Palacios, D. *et al*. The deglaciation of the Americas during the Last Glacial Termination. *Earth-Science Reviews* **203**, 103113, 10.1016/j.earscirev.2020.103113 (2020).

[32] Thackray, G. D., Owen, L. A. & Yi, C. Timing and nature of late Quaternary mountain glaciation. *Journal of Quaternary Science* **23**, 503–508, 10.1002/jqs.1225 (2008).

[33] Haywood, A. M. *et al*. What can Palaeoclimate Modelling do for you? *Earth Systems and Environment* **3**, 1–18, 10.1007/s41748-019-00093-1 (2019).

[34] Schönswetter, P., Stehlik, I., Holderegger, R. & Tribsch, A. Molecular evidence for glacial refugia of mountain plants in the European Alps. *Molecular Ecology* **14**, 3547–3555, 10.1111/j.1365-294X.2005.02683.x (2005). 16156822 10.1111/j.1365-294X.2005.02683.x

[35] Flantua, S. G. A. *et al*. Snapshot isolation and isolation history challenge the analogy between mountains and islands used to understand endemism. *Global Ecology and Biogeography* **29**, 1651–1673, 10.1111/geb.13155 (2020).

[36] Ehlers, J. & Gibbard, P. L. *Quaternary Glaciations: Extent and Chronology*. (Elsevier, Amsterdam, 2004).

[37] *Quaternary Glaciations - Extent and Chronology: A Closer Look*. (Elsevier, Amsterdam; Boston, 2011).

[38] Burkhalter, R. & Bini, A. cartographe. *Die Schweiz während des letzteiszeitlichen Maximums (LGM) = La Suisse durant le dernier maximum glaciaire = La Svizzera durante l’ultimo massimo glaciale = Switzerland during the last glacial maximum*. (Bundesamt für Landestopografie Swisstopo, Wabern, 2009).

[39] Kaufman, D. S., Young, N. E., Briner, J. P. & Manley, W. F. Alaska Palaeo-Glacier Atlas (Version 2). in *Developments in Quaternary Sciences* **15** 427–445. 10.1016/B978-0-444-53447-7.00033-7 (Elsevier, 2011).

[40] Barr, I. D. & Clark, C. D. Late Quaternary glaciations in Far NE Russia; combining moraines, topography and chronology to assess regional and global glaciation synchrony. *Quaternary Science Reviews* **53**, 72–87, 10.1016/j.quascirev.2012.08.004 (2012).

[41] Laabs, B. J. C., Licciardi, J. M., Leonard, E. M., Munroe, J. S. & Marchetti, D. W. Updated cosmogenic chronologies of Pleistocene mountain glaciation in the western United States and associated paleoclimate inferences. *Quaternary Science Reviews* **242**, 106427, 10.1016/j.quascirev.2020.106427 (2020).

[42] *Iberia, Land of Glaciers: How the Mountains Were Shaped by Glaciers*. (Elsevier, Amsterdam, Netherlands; Cambridge, MA, 2022).

[43] Kłapyta, P., Zasadni, J. & Mîndrescu, M. Late Pleistocene glaciation in the Eastern Carpathians – a regional overview. *CATENA* **224**, 106994, 10.1016/j.catena.2023.106994 (2023).

[44] North Dakota State University, Laabs, B., Anderson, L., Licciardi, J. & Tulenko, J. Developing A Geospatial Database of Late Pleistocene Mountain Glaciers in The Western United States. in 387976. 10.1130/abs/2023RM-387976 (2023).

[45] Davies, B. J. *et al*. The evolution of the Patagonian Ice Sheet from 35 ka to the present day (PATICE). *Earth-Science Reviews* **204**, 103152, 10.1016/j.earscirev.2020.103152 (2020).

[46] Dalton, A. S. *et al*. Deglaciation of the north American ice sheet complex in calendar years based on a comprehensive database of chronological data: NADI-1. *Quaternary Science Reviews* **321**, 108345, 10.1016/j.quascirev.2023.108345 (2023).

[47] Stroeven, A. P. *et al*. Deglaciation of Fennoscandia. *Quaternary Science Reviews* **147**, 91–121, 10.1016/j.quascirev.2015.09.016 (2016).

[48] Clark, C. D. *et al*. Growth and retreat of the last British–Irish Ice Sheet, 31 000 to 15 000 years ago: the BRITICE‐CHRONO reconstruction. *Boreas* **51**, 699–758, 10.1111/bor.12594 (2022).

[49] Hendrickx, H., Jacob, M., Frankl, A. & Nyssen, J. Glacial and periglacial geomorphology and its paleoclimatological significance in three North Ethiopian Mountains, including a detailed geomorphological map. *Geomorphology* **246**, 156–167, 10.1016/j.geomorph.2015.05.005 (2015).

[50] Carrasco, R. M., Pedraza, J., Domínguez-Villar, D., Villa, J. & Willenbring, J. K. The plateau glacier in the Sierra de Béjar (Iberian Central System) during its maximum extent. Reconstruction and chronology. *Geomorphology* **196**, 83–93, 10.1016/j.geomorph.2012.03.019 (2013).

[51] Lukas, S. Morphostratigraphic principles in glacier reconstruction -a perspective from the British Younger Dryas. *Progress in Physical Geography: Earth and Environment* **30**, 719–736, 10.1177/0309133306071955 (2006).

[52] Pearce, D., Ely, J., Barr, I. & Boston, C. Chapter 3.4.9 Glacier reconstruction in Geomorphological Techniques. in *Geomorphological Techniques* 1–16 (British Society for Geomorphology, 2017).

[53] James, W. H. M. & Carrivick, J. L. Automated modelling of spatially-distributed glacier ice thickness and volume. *Computers & Geosciences* **92**, 90–103, 10.1016/j.cageo.2016.04.007 (2016).

[54] James, W. H. M., Carrivick, J. L., Quincey, D. J. & Glasser, N. F. A geomorphology based reconstruction of ice volume distribution at the Last Glacial Maximum across the Southern Alps of New Zealand. *Quaternary Science Reviews* **219**, 20–35, 10.1016/j.quascirev.2019.06.035 (2019).

[55] Li, Y. PalaeoIce: An automated method to reconstruct palaeoglaciers using geomorphic evidence and digital elevation models. *Geomorphology* **421**, 108523, 10.1016/j.geomorph.2022.108523 (2023).

[56] Pellitero, R. *et al*. GlaRe, a GIS tool to reconstruct the 3D surface of palaeoglaciers. *Computers & Geosciences* **94**, 77–85, 10.1016/j.cageo.2016.06.008 (2016).

[57] Plummer, M. A. & Phillips, F. M. A 2-D numerical model of snow/ice energy balance and ice flow for paleoclimatic interpretation of glacial geomorphic features. *Quaternary Science Reviews* **22**, 1389–1406, 10.1016/S0277-3791(03)00081-7 (2003).

[58] Leger, T. P. M. *et al*. A data-consistent model of the last glaciation in the Alps achieved with physics-driven AI. *Nature Communications* **16**, 848, 10.1038/s41467-025-56168-3 (2025). 39833153 10.1038/s41467-025-56168-3 PMC11747445

[59] Rocamora, I., Ienco, D. & Ferry, M. Multi-source deep-learning approach for automatic geomorphological mapping: the case of glacial moraines. *Geo-spatial Information Science* **27**, 1747–1766, 10.1080/10095020.2023.2292587 (2024).

[60] Sharp, R. P., Allen, C. R. & Meier, M. F. Pleistocene glaciers on southern California mountains. *American Journal of Science* **257**, 81–94, 10.2475/ajs.257.2.81 (1959).

[61] Sharp, R. P. Pleistocene glaciation in the Trinity Alps of northern California. *American Journal of Science* **258**, 305–340, 10.2475/ajs.258.5.305 (1960).

[62] Emmer, A. *et al*. Glacier retreat and associated processes since the Last Glacial Maximum in the Lejiamayu valley, Peruvian Andes. *Journal of South American Earth Sciences* **109**, 103254, 10.1016/j.jsames.2021.103254 (2021).

[63] Kłapyta, P., Mîndrescu, M. & Zasadni, J. Geomorphological record and equilibrium line altitude of glaciers during the last glacial maximum in the Rodna Mountains (eastern Carpathians). *Quaternary Research* **100**, 1–20, 10.1017/qua.2020.90 (2021).

[64] Kamleitner, S. *et al*. The Ticino-Toce glacier system (Swiss-Italian Alps) in the framework of the Alpine Last Glacial Maximum. *Quaternary Science Reviews* **279**, 107400, 10.1016/j.quascirev.2022.107400 (2022).

[65] Balco, G. Technical note: A prototype transparent-middle-layer data management and analysis infrastructure for cosmogenic-nuclide exposure dating. *Geochronology* **2**, 169–175, 10.5194/gchron-2-169-2020 (2020).

[66] Heyman, J. A global compilation of glacial 10Be and 26Al data. *ExPage (Github Pages)*.

[67] Yokoyama, Y., Lambeck, K., De Deckker, P., Johnston, P. & Fifield, L. K. Timing of the Last Glacial Maximum from observed sea-level minima. *Nature* **406**, 713–716, 10.1038/35021035 (2000). 10963593 10.1038/35021035

[68] Mix, A. Environmental processes of the ice age: land, oceans, glaciers (EPILOG). *Quaternary Science Reviews* **20**, 627–657, 10.1016/S0277-3791(00)00145-1 (2001).

[69] Clark, P. U. & Mix, A. C. Ice sheets and sea level of the Last Glacial Maximum. *Quaternary Science Reviews* **21**, 1–7, 10.1016/S0277-3791(01)00118-4 (2002).

[70] Hughes, P. D., Gibbard, P. L. & Ehlers, J. Timing of glaciation during the last glacial cycle: evaluating the concept of a global ‘Last Glacial Maximum’ (LGM). *Earth-Science Reviews* **125**, 171–198, 10.1016/j.earscirev.2013.07.003 (2013).

[71] Hughes, P. D. & Gibbard, P. L. Evaluating the Concept of a Global “Last Glacial Maximum” (LGM): A Terrestrial Perspective. in *STRATI 2013* (eds. Rocha, R., Pais, J., Kullberg, J. C. & Finney, S.) 943–945. 10.1007/978-3-319-04364-7_177 (Springer International Publishing, Cham, 2014).

[72] Lisiecki, L. E. & Raymo, M. E. A Pliocene‐Pleistocene stack of 57 globally distributed benthic δ ^18^ O records. *Paleoceanography* **20**, 2004PA001071, 10.1029/2004PA001071 (2005).

[73] CLIMAP Project Members. The Surface of the Ice-Age Earth: Quantitative geologic evidence is used to reconstruct boundary conditions for the climate 18,000 years ago. *Science* **191**, 1131–1137, 10.1126/science.191.4232.1131 (1976). 17781630 10.1126/science.191.4232.1131

[74] Cline, R. M. L. *et al*. The Last Interglacial Ocean. *Quaternary Research* **21**, 123–224, 10.1016/0033-5894(84)90098-X (1984).

[75] Peltier, W. R. & Fairbanks, R. G. Global glacial ice volume and Last Glacial Maximum duration from an extended Barbados sea level record. *Quaternary Science Reviews* **25**, 3322–3337, 10.1016/j.quascirev.2006.04.010 (2006).

[76] Hughes, P. D. & Gibbard, P. L. A stratigraphical basis for the Last Glacial Maximum (LGM). *Quaternary International* **383**, 174–185, 10.1016/j.quaint.2014.06.006 (2015).

[77] Shakun, J. D. & Carlson, A. E. A global perspective on Last Glacial Maximum to Holocene climate change. *Quaternary Science Reviews* **29**, 1801–1816, 10.1016/j.quascirev.2010.03.016 (2010).

[78] Smith, J. A., Seltzer, G. O., Farber, D. L., Rodbell, D. T. & Finkel, R. C. Early Local Last Glacial Maximum in the Tropical Andes. *Science* **308**, 678–681, 10.1126/science.1107075 (2005). 15860623 10.1126/science.1107075

[79] Blard, P.-H. *et al*. Late local glacial maximum in the Central Altiplano triggered by cold and locally-wet conditions during the paleolake Tauca episode (17–15ka, Heinrich 1). *Quaternary Science Reviews* **28**, 3414–3427, 10.1016/j.quascirev.2009.09.025 (2009).

[80] Incera Sañudo, L., Rodríguez-Rodríguez, L. & Jiménez-Sánchez, M. Reconstrucción topográfica del paleoglaciar del valle del río Miera (Cantabria) durante el último máximo glaciar local. *Geogaceta* **74**, 51–54, 10.55407/geogaceta98266 (2023).

[81] Clapperton, C. M. Quaternary glaciations in the southern hemisphere: An overview. *Quaternary Science Reviews* **9**, 299–304, 10.1016/0277-3791(90)90024-5 (1990).

[82] Esri. ArcGIS Pro. (2024).

[83] Bini, A. Die Schweiz während des letzteiszeitlichen Maximums (LGM): = La Suisse durant le dernier maximum glaciaire (2009).

[84] Lee, E. *et al*. Palaeoglaciation in the low latitude, low elevation tropical Andes, northern Peru. *Frontiers in Earth Science* **10**, 838826 (2022).

[85] Barrows, T. T., Stone, J. O., Fifield, L. K. & Cresswell, R. G. The timing of the Last Glacial Maximum in Australia. *Quaternary Science Reviews* **21**, 159–173, 10.1016/S0277-3791(01)00109-3 (2002).

[86] agency), W. F. P. (United N. World Administrative Boundaries - Countries and Territories. (2019).

[87] Batchelor, C. L. *et al*. The configuration of Northern Hemisphere ice sheets through the Quaternary. *Nature Communications* **10**, 3713, 10.1038/s41467-019-11601-2 (2019). 31420542 10.1038/s41467-019-11601-2 PMC6697730

[88] Batchelor, C., Manica, A., Murton, D. & Krapp, M. The configuration of Northern Hemisphere ice sheets through the Quaternary. 10.17605/OSF.IO/7JEN3 (2019). 10.1038/s41467-019-11601-2 PMC6697730 31420542

[89] NOAA National Centers for Environmental Information. ETOPO 2022 30 Arc-Second Global Relief Model. 10.25921/FD45-GT74 (2022).

[90] Snethlage, M. A. *et al*. GMBA Mountain Inventory v2: A hierarchical inventory of the world’s mountains for global comparative mountain science. 322mb GMBA-EarthEnv 10.48601/EARTHENV-T9K2-1407 (2021).

[91] Snethlage, M. A. *et al*. A hierarchical inventory of the world’s mountains for global comparative mountain science. *Scientific Data* **9**, 149, 10.1038/s41597-022-01256-y (2022). 35365674 10.1038/s41597-022-01256-y PMC8975823

[92] Benn, D. I. & Hulton, N. R. J. An ExcelTM spreadsheet program for reconstructing the surface profile of former mountain glaciers and ice caps. *Computers & Geosciences* **36**, 605–610, 10.1016/j.cageo.2009.09.016 (2010).

[93] Ferk, M., Gabrovec, M., Komac, B., Zorn, M. & Stepišnik, U. Pleistocene glaciation in Mediterranean Slovenia. *Geological Society, London, Special Publications* **433**, 179–191, 10.1144/SP433.2 (2017).

[94] Kuhle, M. The Last Glacial Maximum (LGM) glacier cover of the Aconcagua group and adjacent massifs in the Mendoza Andes (South America). in *Developments in Quaternary Sciences* **2** 75–82. 10.1016/S1571-0866(04)80113-1 (Elsevier, 2004).

[95] Umer, M., Kebede, S. & Osmaston, H. Quaternary glacial activity on the Ethiopian mountains. in *Developments in Quaternary Sciences* **2** 171–174. 10.1016/S1571-0866(04)80122-2 (Elsevier, 2004).

[96] Lachniet, M. S. & Vazquez-Selem, L. Last Glacial Maximum equilibrium line altitudes in the circum-Caribbean (Mexico, Guatemala, Costa Rica, Colombia, and Venezuela). *Quaternary International* **138–139**, 129–144, 10.1016/j.quaint.2005.02.010 (2005).

[97] Prentice, M. L., Hope, G. S., Peterson, J. A. & Barrows, T. T. The Glaciation of the South-East Asian Equatorial Region. in *Developments in Quaternary Sciences* **15** 1023–1036. 10.1016/B978-0-444-53447-7.00073-8 (Elsevier, 2011).

[98] Serrano, E., González-Trueba, J. J. & González-García, M. Mountain glaciation and paleoclimate reconstruction in the Picos de Europa (Iberian Peninsula, SW Europe). *Quaternary Research* **78**, 303–314, 10.1016/j.yqres.2012.05.016 (2012).

[99] Hannah, G., Hughes, P. D. & Gibbard, P. L. Pleistocene plateau ice fields in the High Atlas, Morocco. *Geological Society, London, Special Publications* **433**, 25–53, 10.1144/SP433.12 (2017).

[100] Pope, R. J. J. *et al*. Long-term glacial and fluvial system coupling in southern Greece and evidence for glaciation during Marine Isotope Stage 16. *Quaternary Science Reviews* **317**, 108239, 10.1016/j.quascirev.2023.108239 (2023).

[101] Heyman, J. *et al*. Palaeoglaciology of Bayan Har Shan, NE Tibetan Plateau: exposure ages reveal a missing LGM expansion. *Quaternary Science Reviews* **30**, 1988–2001, 10.1016/j.quascirev.2011.05.002 (2011).

[102] Lima, C. L. *et al*. Glacimontis. *Zenodo*10.5281/zenodo.15600659 (2025).

[103] Dahms, D. E. Glacial limits in the middle and southern Rocky mountains, U.S.A., south of the Yellowstone ice cap. in *Developments in Quaternary Sciences* **2** 275–288. 10.1016/S1571-0866(04)80203-3 (Elsevier, 2004).

