# Supplementary material for: Mountain glacier extents at the Last Glacial Maximum
Source: Sci Data. 2026 Feb 17;13:629. doi: 10.1038/s41597-026-06841-z (PMC13096163; doi:10.1038/s41597-026-06841-z)
Supplement: Supplementary file 1 — Supplementary Table 1 [file 41597_2026_6841_MOESM1_ESM.doc]

**Supplementary Table 1.** **Attribute table of feature classes of Mountain Paleoglacier Extents**. The worksheet includes the following columns: Field (attribute name), Alias (brief explanation or identifier for the field), Description (further details about the field's purpose or content), Data Type (format of the data, e.g., Numeric), Category (thematic grouping, e.g., Identification for ID numbers, Findings for quantitative results), Feature class (presence of the given field in the feature classes, namely 1.Empirically Reconstructed Paleoglaciers, 2.Reconstruction Constraint Line)

| **Field** | **Alias** | **Description** | **Data Type** | **Category** | **Feature Class** |
| --- | --- | --- | --- | --- | --- |
| OBJECTID | OBJECTID | ESRI ArcGIS Pro’s feature class automated object identifier | Numeric | Identification | 1;2 |
| SHAPE | Shape | ESRI ArcGIS Pro’s feature class automated shape classification | Text | Spatial | 1;2 |
| GlobalID | GlobalID | Automatically generated global shape identifier through the datasets | Text | Identification | 1 |
| mid | Metadata Identifier (MID) | Publication unique identifier related to metadata information | Numeric | Identification | 1; 2 |
| pid | Paleoglacier Identifier (PID) | Study site unique identifier related to paleoglacier and paleoclimate information compiled from literature review | Numeric | Identification | 1 |
| gid | Geographic Identifier (GID) | Spatial identifier of polygon features based on mountain range intersection | Numeric | Identification | 1 |
| reference | Reference | Complete reference of the publication (Nature style) | Text | Referencing | 1 |
| doi | DOI Code | Digital object identifier of the publication | Text | Referencing | 1 |
| scitation | Short Citation | Short citation of the publication | Text | Referencing | 1 |
| yrpubli | Year of Publication | Year the paper or database was published | Numeric | Referencing | 1 |
| addrefs | Additional References | Additional references in the original publication for the glacier reconstructions | Text | Referencing | 1 |
| dataacq | Data Acquisition | Indicates if the reconstruction was digitized from figures or acquired from sources | Text | Referencing | 1 |
| geomap | Geomorphological Mapping | Indicates if a geomorphological map is provided by the referenced publication, where 1 = True and 0 = False | Numeric | Methodology | 1 |
| quantchrono | Quantitative Chronology | Indicates if the referenced study includes chronological data | Numeric | Methodology | 1 |
| cosmodating | Cosmogenic Nuclide Dating | Indicates if cosmogenic nuclide dating methods were used, where 1 = True and 0 = False | Numeric | Methodology | 1 |
| addchrono | Additional Dating Methods | Indicates if other quantitative dating methods were used, where 1 = True and 0 = False | Numeric | Methodology | 1 |
| adddating | Additional Dating Methods Applied | Indicates which additional quantitative dating methods were used by the referenced study/publication | Text | Methodology | 1 |
| mrange1-6 | Mountain Range Level 1-6 | GMBA v2.0 (levels 1-6)[^95^](#Ref76) mountain ranges classification | Text | Spatial | 1 |
| deglalow | Start of Deglaciation Lower Limit (yr BP) | Maximum estimated timing of the LLGM in years, i.e. oldest estimated age | Numeric | Findings | 1 |
| deglalowun | Start of Deglaciation Lower Limit Uncertainty (yr) | Estimated uncertainty for the start of deglaciation lower limit in years | Numeric | Findings | 1 |
| deglaupp | Start of Deglaciation Upper Limit (yr BP) | Minimum estimated timing of the LLGM in years, i.e. youngest estimated age | Numeric | Findings | 1 |
| deglauppun | Start of Deglaciation Upper Limit Uncertainty (ry) | Estimated uncertainty for the start of deglaciation upper limit in years | Numeric | Findings | 1 |
| modela | Modern Equilibrium Line Altitude (m) | Present-day equilibrium-line altitude in meters above present day sea level | Numeric | Findings | 1 |
| paleoela | LLGM Equilibrium Line Altitude (m) | Equilibrium-line altitude during the LLGM in meters above present day sea level | Numeric | Findings | 1 |
| paleoelaun | LLGM Equilibrium Line Altitude Uncertainty (m) | Error margin for the paleo paleo ELA estimate in meters | Numeric | Findings | 1 |
| delamin | Delta Equilibrium Line Altitude Minimum (m) | Difference (lowest value) in meters between present-day and paleo ELA | Numeric | Findings | 1 |
| delamax | Delta Equilibrium Line Altitude Maximum (m) | Difference (highest value) in meters between present-day and paleo ELA | Numeric | Findings | 1 |
| coolmin | Temperature Depression Minimum (°C) | Temperature depression (minimum) from present-day temperature to paleo LLGM temperature in degrees Celsius | Numeric | Findings | 1 |
| coolmax | Temperature Depression Maximum (°C) | Temperature depression (maximum) from present-day temperature to paleo LLGM temperature in degrees Celsius | Numeric | Findings | 1 |
| area | Area (km²) | Total area (km²) in square kilometers of the polygon feature | Numeric | Spatial | 1 |
| perimeter | Perimeter (km) | Total perimeter in kilometers(km) of the polygon feature | Numeric | Spatial | 1; 2 |
| SHAPE_AREA | Area (°²) | ESRI ArcGIS Pro’s feature class automated geometry calculation derived from coordinate system. It represents the total area of the polygon feature in square degrees, a geographic unit based on latitude and longitude. Not visible for GLACIMONTIS purposes | Numeric | Spatial | 1 |
| SHAPE_LENGTH | Perimeter (°) | ESRI ArcGIS Pro’s feature class automated geometry calculation derived from coordinate system. It represents the total area in degrees of the polygon feature. Not visible for GLACIMONTIS purposes | Numeric | Spatial | 1; 2 |
