# Supplementary material for: Mountain glacier extents at the Last Glacial Maximum
Source: Sci Data. 2026 Feb 17;13:629. doi: 10.1038/s41597-026-06841-z (PMC13096163; doi:10.1038/s41597-026-06841-z)
Supplement: Supplementary file 2 — Data Supplementary Information 1 (Data S1) [file 41597_2026_6841_MOESM2_ESM.docx]

# Data Supplementary Information 1

1. Akçar, N. et al. Glacier response to the change in atmospheric circulation in the eastern Mediterranean during the Last Glacial Maximum. Quaternary Geochronology 19, 27–41 (2014). doi: 10.1016/j.quageo.2013.09.004
2. Akçar, N. et al. Synchronous Last Glacial Maximum across the Anatolian peninsula. SP 433, 251–269 (2017). doi: 10.1144/SP433.7
3. Alberti, A. P., Díaz, M. V. & Chao, R. B. Pleistocene glaciation in Spain. in Developments in Quaternary Sciences vol. 2 389–394 (Elsevier, 2004). doi: 10.1016/S1571-0866(04)80089-7
4. Allard, J. L. et al. Late Pleistocene glaciers in Greece: A new 36Cl chronology. Quaternary Science Reviews 245, 106528 (2020). doi: 10.1016/j.quascirev.2020.106528
5. Ammann, C., Jenny, B., Kammer, K. & Messerli, B. Late Quaternary Glacier response to humidity changes in the arid Andes of Chile (18–29°S). Palaeogeography, Palaeoclimatology, Palaeoecology 172, 313–326 (2001). doi: 10.1016/S0031-0182(01)00306-6
6. Ancrenaz, A., Braucher, R., Defive, E., Poiraud, A. & Steiger, J. Last glacial fluctuations in the southwestern Massif Central, Aubrac (France): First direct chronology from cosmogenic 10Be and 26Al exposure dating. Quaternary Science Reviews 285, 107500 (2022). doi: 10.1016/j.quascirev.2022.107500
7. Arkhipov, S. Glaciation of Siberia and north-east USSR. Quaternary Science Reviews 5, 463–474 (1986). doi: 10.1016/0277-3791(86)90212-X
8. Arzhannikov, S. G. et al. History of late Pleistocene glaciations in the central Sayan-Tuva Upland (southern Siberia). Quaternary Science Reviews 49, 16–32 (2012). doi: 10.1016/j.quascirev.2012.06.005
9. Astakhov, V. Pleistocene ice limits in the Russian northern lowlands. in Developments in Quaternary Sciences vol. 2 309–319 (Elsevier, 2004). doi: 10.1016/S1571-0866(04)80081-2
10. Astakhov, V., Shkatova, V., Zastrozhnov, A. & Chuyko, M. Glaciomorphological Map of the Russian Federation. Quaternary International 420, 4–14 (2016). doi: 10.1016/j.quaint.2015.09.024
11. Barnes, D. K. A. et al. Biodiversity signature of the Last Glacial Maximum at South Georgia, Southern Ocean. Journal of Biogeography 43, 2391–2399 (2016). doi: 10.1111/jbi.12855
12. Baroni, C., Guidobaldi, G., Salvatore, M. C., Christl, M. & Ivy-Ochs, S. Last glacial maximum glaciers in the Northern Apennines reflect primarily the influence of southerly storm-tracks in the western Mediterranean. Quaternary Science Reviews 197, 352–367 (2018). doi: 10.1016/j.quascirev.2018.07.003
13. Barr, I. D. & Clark, C. D. Glaciers and climate in Pacific Far NE Russia during the Last Glacial Maximum. J Quaternary Science 26, 227–237 (2011). doi: 10.1002/jqs.1450
14. Barr, I. D. & Clark, C. D. Late Quaternary glaciations in Far NE Russia; combining moraines, topography and chronology to assess regional and global glaciation synchrony. Quaternary Science Reviews 53, 72–87 (2012). doi: 10.1016/j.quascirev.2012.08.004
15. Barrell, D. J. A. Quaternary Glaciers of New Zealand. in Developments in Quaternary Sciences vol. 15 1047–1064 (Elsevier, 2011). doi: 10.1016/B978-0-444-53447-7.00075-1
16. Barrows, T. T., Stone, J. O., Fifield, L. K. & Cresswell, R. G. Late Pleistocene Glaciation of the Kosciuszko Massif, Snowy Mountains, Australia. Quat. res. 55, 179–189 (2001). doi: 10.1006/qres.2001.2216
17. Barrows, T. T., Stone, J. O., Fifield, L. K. & Cresswell, R. G. The timing of the Last Glacial Maximum in Australia. Quaternary Science Reviews 21, 159–173 (2002). doi: 10.1016/S0277-3791(01)00109-3
18. Batchelor, C. L. et al. The configuration of Northern Hemisphere ice sheets through the Quaternary. Nat Commun 10, 3713 (2019). doi: 10.1038/s41467-019-11601-2
19. Bavec, M. & Verbič, T. The extent of Quaternary glaciations in Slovenia. in Developments in Quaternary Sciences vol. 2 385–388 (Elsevier, 2004). doi: 10.1016/S1571-0866(04)80088-5
20. Bayrakdar, C., Çılğın, Z. & Keserci, F. Traces of late quaternary glaciations and paleoclimatic interpretation of Mount Akdağ (Alanya, 2451 m), Southwest Turkey. Med. Geosc. Rev. 2, 135–151 (2020). doi: 10.1007/s42990-020-00026-5
21. Bini, A. Die Schweiz während des letzteiszeitlichen Maximums (LGM): = La Suisse durant le dernier maximum glaciaire [Map]. Swisstopo (2009). doi: 978-3-302-40049-5
22. Bini, A. et al. Glacial history of the southern side of the central Alps, Italy. in Developments in Quaternary Sciences vol. 2 195–200 (Elsevier, 2004). doi: 10.1016/S1571-0866(04)80070-8
23. Blomdin, R. et al. Glacial geomorphology of the Altai and Western Sayan Mountains, Central Asia. Journal of Maps 12, 123–136 (2016). doi: 10.1080/17445647.2014.992177
24. Blomdin, R. et al. Timing and dynamics of glaciation in the Ikh Turgen Mountains, Altai region, High Asia. Quaternary Geochronology 47, 54–71 (2018). doi: 10.1016/j.quageo.2018.05.008
25. Braakhekke, J. et al. Timing and flow pattern of the Orta Glacier (European Alps) during the Last Glacial Maximum. Boreas 49, 315–332 (2020). doi: 10.1111/bor.12427
26. Bromley, G. R. et al. Relative timing of last glacial maximum and late-glacial events in the central tropical Andes. Quaternary Science Reviews 28, 2514–2526 (2009). doi: 10.1016/j.quascirev.2009.05.012
27. Brook, M. S. & Kirkbride, M. P. Reconstruction and paleoclimatic significance of late Quaternary glaciers in the Tararua Range, North Island, New Zealand. Quaternary International 470, 53–66 (2018). doi: 10.1016/j.quaint.2017.10.018
28. Buoncristiani, J.-F. & Campy, M. Palaeogeography of the last two glacial episodes in the Massif Central, France. in Developments in Quaternary Sciences vol. 2 111–112 (Elsevier, 2004). doi: 10.1016/S1571-0866(04)80060-5
29. Buoncristiani, J.-F. & Campy, M. The palaeogeography of the last two glacial episodes in France: the Alps and Jura. in Developments in Quaternary Sciences vol. 2 101–110 (Elsevier, 2004). doi: 10.1016/S1571-0866(04)80059-9
30. Calvet, M., Delmas, M., Gunnell, Y., Braucher, R. & Bourlès, D. Recent Advances in Research on Quaternary Glaciations in the Pyrenees. in Developments in Quaternary Sciences vol. 15 127–139 (Elsevier, 2011). doi: 10.1016/B978-0-444-53447-7.00011-8
31. Campos, N., Palacios, D. & Tanarro, L. M. Glacier reconstruction of La covacha massif in Sierra de Gredos (central Spain) during the last glacial maximum. Journal of Mountain Science 16, 1336–1352 (2019). doi: 10.1007/s11629-019-5382-2
32. Candaş, A., Sarikaya, M. A., Köse, O., Şen, Ö. L. & Çiner, A. Modelling Last Glacial Maximum ice cap with the Parallel Ice Sheet Model to infer palaeoclimate in south‐west Turkey. J Quaternary Science 35, 935–950 (2020). doi: 10.1002/jqs.3239
33. Carraro, F. & Giardino, M. Quaternary glaciations in the western Italian Alps – a review. in Developments in Quaternary Sciences vol. 2 201–208 (Elsevier, 2004). doi: 10.1016/S1571-0866(04)80071-X
34. Carrasco, R. M., Pedraza, J. & Palacios, D. The glaciers of the Sierra de Gredos. in Iberia, Land of Glaciers 457–483 (Elsevier, 2022). doi: 10.1016/B978-0-12-821941-6.00022-0
35. Carrasco, R. M., Pedraza, J. & Palacios, D. The glaciers of the Sierras de Guadarrama and Somosierra. in Iberia, Land of Glaciers 485–503 (Elsevier, 2022). doi: 10.1016/B978-0-12-821941-6.00023-2
36. Carrasco, R. M., Pedraza, J., Domínguez-Villar, D., Villa, J. & Willenbring, J. K. The plateau glacier in the Sierra de Béjar (Iberian Central System) during its maximum extent. Reconstruction and chronology. Geomorphology 196, 83–93 (2013). doi: 10.1016/j.geomorph.2012.03.019
37. Carrasco, R. M., Pedraza, J., Domínguez-Villar, D., Willenbring, J. K. & Villa, J. Sequence and chronology of the Cuerpo de Hombre paleoglacier (Iberian Central System) during the last glacial cycle. Quaternary Science Reviews 129, 163–177 (2015). doi: 10.1016/j.quascirev.2015.09.021
38. Castiglioni, G. B. Quaternary glaciations in the eastern sector of the Italian Alps. in Developments in Quaternary Sciences vol. 2 209–214 (Elsevier, 2004). doi: 10.1016/S1571-0866(04)80072-1
39. Ceballos Liévano, J.L., Rodríguez Murcia, C.E., Real Núñez, E.L. (Eds.). Glaciares de Colombia: más que montañas con hielo. IDEAM, Instituto de Hidrología Meteorología y Estudios Ambientales, Bogotá (2012). doi: 978-958-8067-57-5
40. Chen, A., Wang, N., Guo, Z., Wu, Y. & Wu, H. Glacier variations and rising temperature in the Mt. Kenya since the Last Glacial Maximum. J. Mt. Sci. 15, 1268–1282 (2018). doi: 10.1007/s11629-017-4600-z
41. Çilğin, Z. 3D Surface Modeling of Late Pleistocene Glaciers in the Munzur Mountains (Eastern Turkey) and its paleoclimatic implications. Turkish J Earth Sci 29, 714–732 (2020). doi: 10.3906/yer-1905-18
42. Clapperton, C. M. The glaciation of the Andes. Quaternary Science Reviews 2, 83–155 (1983). doi: 10.1016/0277-3791(83)90005-7
43. Coggan, B. & Burenjargal, U. Glaciations of the Davaatiin Region of the Hangay Nuruu, Central Mongolia. 20th Annual Keck Symposium (2007).
44. Colhoun, E. A. & Barrows, T. T. The Glaciation of Australia. in Developments in Quaternary Sciences vol. 15 1037–1045 (Elsevier, 2011). doi: 10.1016/B978-0-444-53447-7.00074-X
45. Cowton, T., Hughes, P. D. & Gibbard, P. L. Palaeoglaciation of Parque Natural Lago de Sanabria, northwest Spain. Geomorphology 108, 282–291 (2009). doi: 10.1016/j.geomorph.2009.02.007
46. Dahms, D. E. Glacial limits in the middle and southern Rocky mountains, U.S.A., south of the Yellowstone ice cap. in Developments in Quaternary Sciences vol. 2 275–288 (Elsevier, 2004). doi: 10.1016/S1571-0866(04)80203-3
47. Dahms, D. E. Glacial limits in the middle and southern Rocky mountains, U.S.A., south of the Yellowstone ice cap. in Developments in Quaternary Sciences vol. 2 275–288 (Elsevier, 2004). doi: 10.1016/S1571-0866(04)80203-3
48. Davies, B. J. et al. The evolution of the Patagonian Ice Sheet from 35 ka to the present day (PATICE). Earth-Science Reviews 204, 103152 (2020). doi: 10.1016/j.earscirev.2020.103152
49. Deline, P. et al. Timing and extent of glaciation since the Last Glacial Maximum in the upper Kalguty basin, Russian Altai Mountains. J Quaternary Science 38, 1082–1102 (2023). doi: 10.1002/jqs.3535
50. Delmas, M., Gunnell, Y., Calvet, M., Reixach, T. & Oliva, M. The Pyrenees: glacial landforms prior to the Last Glacial Maximum. in European Glacial Landscapes 295–307 (Elsevier, 2022). doi: 10.1016/B978-0-12-823498-3.00035-2
51. Dong, G. et al. Cosmogenic 10 Be surface exposure dating and glacier reconstruction for the Last Glacial Maximum in the Quemuqu Valley, western Nyainqentanglha Mountains, south Tibet. J Quaternary Science 32, 639–652 (2017). doi: 10.1002/jqs.2963
52. Ehlers, J., Gibbard, P. L. & Hughes, P. D. Introduction. in Developments in Quaternary Sciences vol. 15 1–14 (Elsevier, 2011). doi: 10.1016/B978-0-444-53447-7.00001-5
53. Ehlers, J., Grube, A., Stephan, H.-J. & Wansa, S. Pleistocene Glaciations of North Germany—New Results. in Developments in Quaternary Sciences vol. 15 149–162 (Elsevier, 2011). doi: 10.1016/B978-0-444-53447-7.00013-1
54. Emmer, A. et al. Glacier retreat and associated processes since the Last Glacial Maximum in the Lejiamayu valley, Peruvian Andes. Journal of South American Earth Sciences 109, 103254 (2021). doi: 10.1016/j.jsames.2021.103254
55. Ferk, M., Gabrovec, M., Komac, B., Zorn, M. & Stepišnik, U. Pleistocene glaciation in Mediterranean Slovenia. SP 433, 179–191 (2017). doi: 10.1144/SP433.2
56. Fiebig, M., Buiter, S. J. H. & Ellwanger, D. Pleistocene glaciations of South Germany. in Developments in Quaternary Sciences vol. 2 147–154 (Elsevier, 2004). doi: 10.1016/S1571-0866(04)80065-4
57. Figueira, E., Gomes, A. & Pérez-Alberti, A. Pleistocene Glaciations of the Northwest of Iberia: Glacial Maximum Extent, Ice Thickness, and ELA of the Soajo Mountain. Land 12, 1226 (2023). doi: 10.3390/land12061226
58. Gallardo, M., Otto, J.-C., Gayo, E. M. & Sitzia, L. Reconstruction of glaciers in the western boundary of the Altiplano (18.5°-19°S): Singularities and insights on potential drivers of past advances. Quaternary Science Advances 13, 100158 (2024). doi: 10.1016/j.qsa.2023.100158
59. Ganyushkin, D. et al. Palaeoclimate, glacier and treeline reconstruction based on geomorphic evidences in the Mongun-Taiga massif (south-eastern Russian Altai) during the Late Pleistocene and Holocene. Quaternary International 470, 26–37 (2018). doi: 10.1016/j.quaint.2017.12.031
60. García-Ruiz, J. M. The glaciers of the Iberian Range. in Iberia, Land of Glaciers 437–455 (Elsevier, 2022)-9. doi: 10.1016/B978-0-12-821941-6.00021-9
61. Gillespie, A. R. & Zehfuss, P. H. Glaciations of the Sierra Nevada, California, USA. in Developments in Quaternary Sciences vol. 2 51–62 (Elsevier, 2004). doi: 10.1016/S1571-0866(04)80185-4
62. Gillespie, A. R. & Zehfuss, P. H. Glaciations of the Sierra Nevada, California, USA. in Developments in Quaternary Sciences vol. 2 51–62 (Elsevier, 2004).. doi: 10.1016/S1571-0866(04)80185-4
63. Giraudi, C. Middle Pleistocene to Holocene Glaciations in the Italian Apennines. in Developments in Quaternary Sciences vol. 15 211–219 (Elsevier, 2011). doi: 10.1016/B978-0-444-53447-7.00017-9
64. Giraudi, C. The Upper Pleistocene deglaciation on the Apennines (Peninsular Italy). CIG 41, 337–358 (2015). doi: 10.18172/cig.2696
65. Gobejishvili, R., Lomidze, N. & Tielidze, L. Late Pleistocene (Würmian) Glaciations of the Caucasus. in Developments in Quaternary Sciences vol. 15 141–147 (Elsevier, 2011). doi: 10.1016/B978-0-444-53447-7.00012-X
66. Gómez-Ortiz, A., Oliva, M., Palacios, D. & Salvador-Franch, F. The glaciers of the Sierra Nevada. in Iberia, Land of Glaciers 505–524 (Elsevier, 2022). doi: 10.1016/B978-0-12-821941-6.00024-4
67. Gómez-Ortiz, A., Palacios, D., Palade, B., Vázquez-Selem, L. & Salvador-Franch, F. The deglaciation of the Sierra Nevada (Southern Spain). Geomorphology 159–160, 93–105 (2012). doi: 10.1016/j.geomorph.2012.03.008
68. Groos, A. R. et al. Nonuniform Late Pleistocene glacier fluctuations in tropical Eastern Africa. Sci. Adv. 7, eabb6826 (2021). doi: 10.1126/sciadv.abb6826
69. Grunert, J., Lehmkuhl, F. & Walther, M. Paleoclimatic evolution of the Uvs Nuur basin and adjacent areas (Western Mongolia). Quaternary International 65–66, 171–192 (2000). doi: 10.1016/S1040-6182(99)00043-9
70. Hall, K. & Meiklejohn, I. Glaciation in Southern Africa and in the Sub-Antarctic. in Developments in Quaternary Sciences vol. 15 1081–1085 (Elsevier, 2011). doi: 10.1016/B978-0-444-53447-7.00078-7
71. Hall, K. Quaternary glaciation of the sub-Antarctic Islands. in Developments in Quaternary Sciences vol. 2 339–345 (Elsevier, 2004). doi: 10.1016/S1571-0866(04)80140-4
72. Hannah, G., Hughes, P. D. & Gibbard, P. L. Pleistocene plateau ice fields in the High Atlas, Morocco. SP 433, 25–53 (2017). doi: 10.1144/SP433.12
73. Heine, K. Late Quaternary Glaciations in Bolivia: Comments on Some New Approaches to Dating Morainic Sequences. in Developments in Quaternary Sciences vol. 15 757–772 (Elsevier, 2011). doi: 10.1016/B978-0-444-53447-7.00055-6
74. Hendrickx, H., Jacob, M., Frankl, A. & Nyssen, J. Glacial and periglacial geomorphology and its paleoclimatological significance in three North Ethiopian Mountains, including a detailed geomorphological map. Geomorphology 246, 156–167 (2015). doi: 10.1016/j.geomorph.2015.05.005
75. Hofmann, F. M., Rauscher, F., McCreary, W., Bischoff, J.-P. & Preusser, F. Revisiting Late Pleistocene glacier dynamics north-west of the Feldberg, southern Black Forest, Germany. E&amp;G Quaternary Sci. J. 69, 61–87 (2020). doi: 10.5194/egqsj-69-61-2020
76. Hughes, P. D., Woodward, J. C., Van Calsteren, P. C. & Thomas, L. E. The glacial history of the Dinaric Alps, Montenegro. Quaternary Science Reviews 30, 3393–3412 (2011). doi: 10.1016/j.quascirev.2011.08.016
77. Hughes, P. D., Woodward, J. C., Van Calsteren, P. C., Thomas, L. E. & Adamson, K. R. Pleistocene ice caps on the coastal mountains of the Adriatic Sea. Quaternary Science Reviews 29, 3690–3708 (2010). doi: 10.1016/j.quascirev.2010.06.032
78. Ignéczi, Á. & Nagy, B. Former plateau ice fields in the Godeanu Mountains, Southern Carpathians: First evidence of glaciated peneplains in the Carpathians. Quaternary International 415, 74–85 (2016). doi: 10.1016/j.quaint.2015.09.058
79. Incera Sañudo, L., Rodríguez-Rodríguez, L. & Jiménez-Sánchez, M. Reconstrucción topográfica del paleoglaciar del valle del río Miera (Cantabria) durante el último máximo glaciar local. Geogaceta 74, 51–54 (2023). doi: 10.55407/geogaceta98266
80. James, W. H. M., Carrivick, J. L., Quincey, D. J. & Glasser, N. F. A geomorphology based reconstruction of ice volume distribution at the Last Glacial Maximum across the Southern Alps of New Zealand. Quaternary Science Reviews 219, 20–35 (2019). doi: 10.1016/j.quascirev.2019.06.035
81. Jiménez Sánchez, M. El glaciarismo en la cuenca alta del río Nalón (NO de España): una propuesta de evolución de los sistemas glaciares cuaternarios en la Cordillera Cantábrica. Revista de la Sociedad Geológica de España 9, 157–168 (1996). doi:
82. Jiménez-Sánchez, M., Rodríguez-Rodríguez, L., González-Lemos, S. & Domínguez-Cuesta, M. J. The glaciers in the Redes Natural Park. in Iberia, Land of Glaciers 221–235 (Elsevier, 2022). doi: 10.1016/B978-0-12-821941-6.00011-6
83. Kamleitner, S. et al. The Ticino-Toce glacier system (Swiss-Italian Alps) in the framework of the Alpine Last Glacial Maximum. Quaternary Science Reviews 279, 107400 (2022). doi: 10.1016/j.quascirev.2022.107400
84. Kamp, U. & Haserodt, K. Quaternary glaciations in the high mountains of northern Pakistan. in Developments in Quaternary Sciences vol. 2 293–311 (Elsevier, 2004). doi: 10.1016/S1571-0866(04)80135-0
85. Kaufman, D. S., Young, N. E., Briner, J. P. & Manley, W. F. Alaska Palaeo-Glacier Atlas (Version 2). in Developments in Quaternary Sciences vol. 15 427–445 (Elsevier, 2011). doi: 10.1016/B978-0-444-53447-7.00033-7
86. Keserci, F., Bayrakdar, C., Çılğın, Z. & Evans, I. S. Modeling the form, distribution and paleoclimatic implications of former glaciers in the Teke Peninsula (Eastern Mediterranean, Turkey). Geomorphology 431, 108683 (2023). doi: 10.1016/j.geomorph.2023.108683
87. Kłapyta, P., Mîndrescu, M. & Zasadni, J. Geomorphological record and equilibrium line altitude of glaciers during the last glacial maximum in the Rodna Mountains (eastern Carpathians). Quat. res. 100, 1–20 (2021). doi: 10.1017/qua.2020.90
88. Kłapyta, P., Zasadni, J. & Mîndrescu, M. Late Pleistocene glaciation in the Eastern Carpathians – a regional overview. CATENA 224, 106994 (2023). doi: 10.1016/j.catena.2023.106994
89. Klein, A. G., Seltzer, G. O. & Isacks, B. L. Modern and last local glacial maximum snowlines in the Central Andes of Peru, Bolivia, and Northern Chile. Quaternary Science Reviews 18, 63–84 (1999). doi: 10.1016/j.quaint.2005.02.012
90. Kuhle, M. The High Glacial (Last Ice Age and Last Glacial Maximum) Ice Cover of High and Central Asia, with a Critical Review of Some Recent OSL and TCN Dates. in Developments in Quaternary Sciences vol. 15 943–965 (Elsevier, 2011). doi: 10.1016/B978-0-444-53447-7.00068-4
91. Kuhle, M. The Pleistocene Glaciation (LGP and pre-LGP, pre-LGM) of SE Iranian Mountains Exemplified by the Kuh-i-Jupar, Kuh-i-Lalezar and Kuh-i-Hezar Massifs in the Zagros. Polarforschung 18 pages (2008). doi: 10.2312/POLARFORSCHUNG.77.2-3.71
92. Kuhlemann, J. et al. Glaciation in the Rila Mountains (Bulgaria) during the last glacial maximum. Quaternary International 293, 51–62 (2013). doi: 10.1016/j.quaint.2012.06.027
93. Kuhlemann, J., Frisch, W., Szekely, B., Dunkl, I, Danisik, M & Krumrei, I. Würmian maximum glaciation in Corsica. Austrian Journal of Earth Sciences 97, 68 - 81 (2004). doi:
94. Kuhlemann, J., Milivojevič M., Krumrei, I. and Kubik, P.W. Last glaciation of the Šara Range (Balkan peninsula): Increasing dryness from the LGM to the Holocene. Austrian Journal of Earth Sciences 102, 146-158 (2009). doi:
95. Kull, C., Imhof, S., Grosjean, M., Zech, R. & Veit, H. Late Pleistocene glaciation in the Central Andes: Temperature versus humidity control — A case study from the eastern Bolivian Andes (17°S) and regional synthesis. Global and Planetary Change 60, 148–164 (2008). doi: 10.1016/j.gloplacha.2007.03.011
96. Kumar, P. et al. Last Glacial Maximum and subsequent glacial chronology in the monsoon-dominated Sikkim Himalaya, India. Palaeogeography, Palaeoclimatology, Palaeoecology 617, 111480 (2023). doi: 10.1016/j.palaeo.2023.111480
97. La Frenierre, J., Huh, K. I. & Mark, B. G. Ecuador, Peru and Bolivia. in Developments in Quaternary Sciences vol. 15 773–802 (Elsevier, 2011). doi: 10.1016/B978-0-444-53447-7.00056-8
98. Laabs, B. J. C. & Munroe, J. S. Glacial geologic map of the Uinta Mountains area, Utah and Wyoming. (2009). doi:
99. Laabs, B. J. C. et al. Chronology of latest Pleistocene mountain glaciation in the western Wasatch Mountains, Utah, U.S.A. Quat. res. 76, 272–284 (2011). doi: 10.1016/j.yqres.2011.06.016
100. Lachniet, M. S. & Roy, A. J. Costa Rica and Guatemala. in Developments in Quaternary Sciences vol. 15 843–848 (Elsevier, 2011). doi: 10.1016/B978-0-444-53447-7.00060-X
101. Lachniet, M. S. & Seltzer, G. O. Late Quaternary glaciation of Costa Rica. Geological Society of America Bulletin 114, 547–558 (2002). doi: 10.1130/0016-7606(2002)114<0547:LQGOCR>2.0.CO;2
102. Lachniet, M. S. & Vazquez-Selem, L. Last glacial maximum equilibrium line altitudes in the circum-Caribbean (Mexico, Guatemala, Costa Rica, Colombia, and Venezuela). Quaternary International 138, 129–144 (2005). doi: 10.1016/j.quaint.2005.02.010
103. László, P., Kern, Z. & Nagy, B. Late Pleistocene glaciers in the western Rodna Mountains, Romania. Quaternary International 293, 79–91 (2013). doi: 10.1016/j.quaint.2012.09.020
104. Lee, E. et al. Palaeoglaciation in the low latitude, low elevation tropical Andes, northern Peru. Frontiers in Earth Science 10, 838826 (2022). doi: 10.3389/feart.2022.838826
105. Lehmkuhl, F., Klinge, M. & Stauch, G. The Extent and Timing of Late Pleistocene Glaciations in the Altai and Neighbouring Mountain Systems. in Developments in Quaternary Sciences vol. 15 967–979 (Elsevier, 2011). doi: 10.1016/B978-0-444-53447-7.00069-6
106. Leontaritis, A. D., Kouli, K. & Pavlopoulos, K. The glacial history of Greece: a comprehensive review. Mediterranean Geoscience Reviews 2, 65–90 (2020). doi: 10.1007/s42990-020-00021-w
107. Licciardi, J. M. & Pierce, K. L. History and dynamics of the Greater Yellowstone Glacial System during the last two glaciations. Quaternary Science Reviews 200, 1–33 (2018). doi: 10.1016/j.quascirev.2018.08.027
108. Liu, J. et al. Glacier changes since the Last Glacial Maximum on two slopes of Mt Noijin Kang‐Sang, Southern Tibetan Plateau. J Quaternary Science 39, 70–81 (2024). doi: 10.1002/jqs.3569
109. Locke, W. & Smith, L. N. Pleistocene mountain glaciation in Montana, USA. in Developments in Quaternary Sciences vol. 2 125–129 (Elsevier, 2004). doi: 10.1016/S1571-0866(04)80193-3
110. Locke, W. & Smith, L. N. Pleistocene mountain glaciation in Montana, USA. in Developments in Quaternary Sciences vol. 2 125–129 (Elsevier, 2004). doi: 10.1016/S1571-0866(04)80193-3
111. Mackintosh, A. N., Barrows, T. T., Colhoun, E. A. & Fifield, L. K. Exposure dating and glacial reconstruction at Mt. Field, Tasmania, Australia, identifies MIS 3 and MIS 2 glacial advances and climatic variability. J Quaternary Science 21, 363–376 (2006). doi: 10.1002/jqs.989
112. Makos, M. et al. Last Glacial Maximum and Lateglacial in the Polish High Tatra Mountains - Revised deglaciation chronology based on the 10Be exposure age dating. Quaternary Science Reviews 187, 130–156 (2018). doi: 10.1016/j.quascirev.2018.03.006
113. Makos, M., Dzierżek, J., Nitychoruk, J. & Zreda, M. Timing of glacier advances and climate in the High Tatra Mountains (Western Carpathians) during the Last Glacial Maximum. Quat. res. 82, 1–13 (2014). doi: 10.1016/j.yqres.2014.04.001
114. Margold, M. et al. Extensive glaciation in Transbaikalia, Siberia, at the Last Glacial Maximum. Quaternary Science Reviews 132, 161–174 (2016). doi: 10.1016/j.quascirev.2015.11.018
115. Marjanac, L. & Marjanac, T. Glacial history of the Croatian Adriatic and Coastal Dinarides. in Developments in Quaternary Sciences vol. 2 19–26 (Elsevier, 2004). doi: 10.1016/S1571-0866(04)80053-8
116. Mark, B. G. & Helmens, K. F. Reconstruction of glacier equilibrium‐line altitudes for the Last Glacial Maximum on the High Plain of Bogotá, Eastern Cordillera, Colombia: climatic and topographic implications. J Quaternary Science 20, 789–800 (2005). doi: 10.1002/jqs.974
117. Mark, B. G., Seltzer, G. O., Rodbell, D. T. & Goodman, A. Y. Rates of Deglaciation during the Last Glaciation and Holocene in the Cordillera Vilcanota-Quelccaya Ice Cap Region, Southeastern Perú. Quat. res. 57, 287–298 (2002). doi: 10.1006/qres.2002.2320
118. Martini, M. A. et al. Late Pleistocene glacial fluctuations in Cordillera oriental, subtropical Andes. Quaternary Science Reviews 171, 245–259 (2017). doi: 10.1016/j.quascirev.2017.06.033
119. McCarthy, A., Mackintosh, A., Rieser, U. & Fink, D. Mountain Glacier Chronology from Boulder Lake, New Zealand, Indicates MIS 4 and MIS 2 Ice Advances of Similar Extent. Arctic, Antarctic, and Alpine Research 40, 695–708 (2008). doi: 10.1657/1523-0430(06-111)[MCCARTHY]2.0.CO;2
120. Menkovic, L. et al. Glacial morphology of Serbia, with comments on the Pleistocene Glaciation of Monte Negro, Macedonia and Albania. in Developments in Quaternary Sciences vol. 2 379–384 (Elsevier, 2004). doi: 10.1016/S1571-0866(04)80087-3
121. Mercier, J.-L. & Jeser, N. The glacial history of the Vosges Mountains. in Developments in Quaternary Sciences vol. 2 113–118 (Elsevier, 2004). doi: 10.1016/S1571-0866(04)80061-7
122. Milivojević, M., Menković, L. & Ćalić, J. Pleistocene glacial relief of the central part of Mt. Prokletije (Albanian Alps). Quaternary International 190, 112–122 (2008). doi: 10.1016/j.quaint.2008.04.006
123. Moore, E. M. M. et al. Climate reconstructions for the Last Glacial Maximum from a simple cirque glacier in Fiordland, New Zealand. Quaternary Science Reviews 275, 107281 (2022). doi: 10.1016/j.quascirev.2021.107281
124. Moulin, A. et al. LGM glaciers in the SE Mediterranean? First evidence from glacial landforms and 36Cl dating on Mount Lebanon. Quaternary Science Reviews 285, 107502 (2022). doi: 10.1016/j.quascirev.2022.107502
125. Nieto, C. E. et al. Spatial and Paleoclimatic Reconstruction of the Peña Negra Paleoglacier (Sierra de Béjar-Candelario, Spain) during the Last Glacial Cycle (Late Pleistocene). Sustainability 15, 16514 (2023). doi: 10.3390/su152316514
126. North Dakota State University, Laabs, B., Anderson, L., Licciardi, J. & Tulenko, J. DEVELOPING A GEOSPATIAL DATABASE OF LATE PLEISTOCENE MOUNTAIN GLACIERS IN THE WESTERN UNITED STATES. in 387976 (2023). doi: 10.1130/abs/2023RM-387976
127. Nývlt, D., Engel, Z. & Tyráček, J. Pleistocene Glaciations of Czechia. in Developments in Quaternary Sciences vol. 15 37–46 (Elsevier, 2011). doi: 10.1016/B978-0-444-53447-7.00004-0
128. Ono, Y., Aoki, T., Hasegawa, H. & Dali, L. Mountain glaciation in Japan and Taiwan at the global Last Glacial Maximum. Quaternary International 138–139, 79–92 (2005). doi: 10.1016/j.quaint.2005.02.007
129. Osborn, G. Great Basin of the western United States. in Developments in Quaternary Sciences vol. 2 63–67 (Elsevier, 2004). doi: 10.1016/S1571-0866(04)80186-6
130. Osborn, G. Great Basin of the western United States. in Developments in Quaternary Sciences vol. 2 63–67 (Elsevier, 2004). doi: 10.1016/S1571-0866(04)80186-6
131. Osipov, E. Y. Equilibrium-line altitudes on reconstructed LGM glaciers of the northwest Barguzinsky Ridge, Northern Baikal, Russia. Palaeogeography, Palaeoclimatology, Palaeoecology 209, 219–226 (2004). doi: 10.1016/j.palaeo.2004.02.021
132. Osmaston, H. A., Mitchell, W. A. & Osmaston, J. A. N. Quaternary glaciation of the Bale Mountains, Ethiopia. J Quaternary Science 20, 593–606 (2005). doi: 10.1002/jqs.931
133. Osmaston, H. Quaternary glaciations in the East African mountains. in Developments in Quaternary Sciences vol. 2 139–150 (Elsevier, 2004). doi: 10.1016/S1571-0866(04)80119-2
134. Owen, L. A. & Benn, D. I. Equilibrium-line altitudes of the Last Glacial Maximum for the Himalaya and Tibet: an assessment and evaluation of results. Quaternary International 138–139, 55–78 (2005). doi: 10.1016/j.quaint.2005.02.006
135. Owen, L. A. Quaternary Glaciation of Northern India. in Developments in Quaternary Sciences vol. 15 929–942 (Elsevier, 2011). doi: 10.1016/B978-0-444-53447-7.00067-2
136. Paul, O. J., Dar, R. A. & Romshoo, S. A. Paleo-glacial and paleo-equilibrium line altitude reconstruction from the Late Quaternary glacier features in the Pir Panjal Range, NW Himalayas. Quaternary International 642, 5–16 (2022). doi: 10.1016/j.quaint.2021.03.005
137. Pedraza, J., Carrasco, R. M., Domínguez-Villar, D. & Villa, J. Late Pleistocene glacial evolutionary stages in the Gredos Mountains (Iberian Central System). Quaternary International 302, 88–100 (2013). doi: 10.1016/j.quaint.2012.10.038
138. Pellitero, R. The glaciers of the Montaña Palentina. in Iberia, Land of Glaciers 179–199 (Elsevier, 2022). doi: 10.1016/B978-0-12-821941-6.00009-8
139. Pellitero, R., Fernández‐Fernández, J. M., Campos, N., Serrano, E. & Pisabarro, A. Late Pleistocene climate of the northern Iberian Peninsula: New insights from palaeoglaciers at Fuentes Carrionas (Cantabrian Mountains). J Quaternary Science 34, 342–354 (2019). doi: 10.1002/jqs.3106
140. Pérez-Alberti, A. & Gómez-Pazo, A. Glaciers Landscapes during the Pleistocene in Trevinca Massif (Northwest Iberian Peninsula). Land 12, 530 (2023). doi: 10.3390/land12030530
141. Pérez-Alberti, A. & Valcarcel, M. The glaciers in Eastern Galicia. in Iberia, Land of Glaciers 375–395 (Elsevier, 2022). doi: 10.1016/B978-0-12-821941-6.00018-9
142. Pérez-Alberti, A. The glaciers of the Peneda, Amarela, and Gerês-Xurés massifs. in Iberia, Land of Glaciers 397–416 (Elsevier, 2022). doi: 10.1016/B978-0-12-821941-6.00019-0
143. Peterson, J. A., Chandra, S. & Lundberg, C. Landforms from the Quaternary glaciation of Papua New Guinea: an overview of ice extent during the Last Glacial Maximum. in Developments in Quaternary Sciences vol. 2 313–319 (Elsevier, 2004). doi: 10.1016/S1571-0866(04)80136-2
144. Petrović, A. S. A Reconstruction of the Pleistocene Glacial Maximum in the Žijovo Range (Prokletije Mountains, Montenegro). AGS 54, (2014). doi: 10.3986/AGS54202
145. Pope, R. J. J. et al. Long-term glacial and fluvial system coupling in southern Greece and evidence for glaciation during Marine Isotope Stage 16. Quaternary Science Reviews 317, 108239 (2023). doi: 10.1016/j.quascirev.2023.108239
146. Porter, S. C. Glaciation of western washington, U.S.A. in Developments in Quaternary Sciences vol. 2 289–293 (Elsevier, 2004). doi: 10.1016/S1571-0866(04)80204-5
147. Porter, S. C. Late Pleistocene Glaciation of the Hindu Kush, Afghanistan. in Developments in Quaternary Sciences vol. 15 863–864 (Elsevier, 2011). doi: 10.1016/B978-0-444-53447-7.00062-3
148. Porter, S. C. Pleistocene snowlines and glaciation of the Hawaiian Islands. Quaternary International 138–139, 118–128 (2005). doi: 10.1016/j.quaint.2005.02.009
149. Prentice, M. L., Hope, G. S., Maryunani, K. & Peterson, J. A. An evaluation of snowline data across New Guinea during the last major glaciation, and area-based glacier snowlines in the Mt. Jaya region of Papua, Indonesia, during the Last Glacial Maximum. Quaternary International 138–139, 93–117 (2005). doi: 10.1016/j.quaint.2005.02.008
150. Prentice, M. L., Hope, G. S., Peterson, J. A. & Barrows, T. T. The Glaciation of the South-East Asian Equatorial Region. in Developments in Quaternary Sciences vol. 15 1023–1036 (Elsevier, 2011). doi: 10.1016/B978-0-444-53447-7.00073-8
151. Pyrda, A. Glacial geomorphology and Pleistocene glacier reconstruction in the Demänovská Valley, Low Tatra Mountains, Slovakia. geol 49, 19–36 (2023). doi: 10.7494/geol.2023.49.1.19
152. R. Schuster, A. D., H. G. Krenmayr, M. Linner, G. W. Mandl, G. Pestal &. J. M. Reitner. Rocky Austria: Geologie von Österreich Kurz Und Bunt. (Geologische Bundesanstalt, 2020). doi: 978-3-903252-00-4
153. Ramage, J. M., Smith, J. A., Rodbell, D. T. & Seltzer, G. O. Comparing reconstructed Pleistocene equilibrium‐line altitudes in the tropical Andes of central Peru. J Quaternary Science 20, 777–788 (2005). doi: 10.1002/jqs.982
154. Reber, R. et al. Glacier advances in northeastern Turkey before and during the global Last Glacial Maximum. Quaternary Science Reviews 101, 177–192 (2014). doi: 10.1016/j.quascirev.2014.07.014
155. Reber, R. et al. LGM glaciations in the northeastern anatolian mountains: new insights. Geosciences 12, 257 (2022). doi: 10.3390/geosciences12070257
156. Redondo-Vega, J. M., Santos-González, J., González-Gutiérrez, R. B. & Gómez-Villar, A. The glaciers of the Montes de León. in Iberia, Land of Glaciers 315–333 (Elsevier, 2022). doi: 10.1016/B978-0-12-821941-6.00015-3
157. Refsnider, K. A., Brugger, K. A., Leonard, E. M., McCALPIN, J. P. & Armstrong, P. P. Last Glacial Maximum equilibrium‐line altitude trends and precipitation patterns in the Sangre de Cristo Mountains, southern Colorado, USA. Boreas 38, 663–678 (2009). doi: 10.1111/j.1502-3885.2009.00097.x
158. Rettig, L. et al. Responses of small mountain glaciers in the Maritime Alps (south-western European Alps) to climatic changes during the Last Glacial Maximum. Quaternary Science Reviews 325, 108484 (2024). doi: 10.1016/j.quascirev.2023.108484
159. Rettig, L., Monegato, G., Spagnolo, M., Hajdas, I. & Mozzi, P. The Equilibrium Line Altitude of isolated glaciers during the Last Glacial Maximum – New insights from the geomorphological record of the Monte Cavallo Group (south-eastern European Alps). CATENA 229, 107187 (2023). doi: 10.1016/j.catena.2023.107187
160. Reuther, A. U. et al. Late Pleistocene glacial chronology of the Pietrele Valley, Retezat Mountains, Southern Carpathians constrained by 10Be exposure ages and pedological investigations. Quaternary International 164–165, 151–169 (2007). doi: 10.1016/j.quaint.2006.10.011
161. Ribolini, A., Isola, I., Zanchetta, G., Bini, M. & Sulpizio, R. Glacial features on the Galicica Mountains, Macedonia, Preliminary report. Geogr. Fis. Din. Quat 34, 247–255 (2011). doi: 10.4461/GFDQ.2011.34.22
162. Rodríguez-Rodríguez, L., Jiménez-Sánchez, M., Domínguez-Cuesta, M. J. & González-Lemos, S. The glaciers around Lake Sanabria. in Iberia, Land of Glaciers 335–351 (Elsevier, 2022). doi: 10.1016/B978-0-12-821941-6.00016-5
163. Rother, H., Lehmkuhl, F., Fink, D. & Nottebaum, V. Surface exposure dating reveals MIS-3 glacial maximum in the Khangai Mountains of Mongolia. Quat. res. 82, 297–308 (2014). doi: 10.1016/j.yqres.2014.04.006
164. Rother, H., Shulmeister, J., Fink, D., Alexander, D. & Bell, D. Surface exposure chronology of the Waimakariri glacial sequence in the Southern Alps of New Zealand: Implications for MIS-2 ice extent and LGM glacial mass balance. Earth and Planetary Science Letters 429, 69–81 (2015). doi: 10.1016/j.epsl.2015.07.033
165. Roy, A. J. & Lachniet, M. S. Late quaternary glaciation and equilibrium-line altitudes of the mayan ice cap, Guatemala, Central America. Quaternary Research 74, 1–7 (2010). doi: 10.1016/j.yqres.2010.04.010
166. Ruiz-Fernández, J., García-Hernández, C. & Gallinar Cañedo, D. The glaciers of the Picos de Europa. in Iberia, Land of Glaciers 237–263 (Elsevier, 2022). doi: 10.1016/B978-0-12-821941-6.00012-8
167. Ruiz-Fernández, J., González-Díaz, B., Cañedo, D. G. & García-Hernández, C. The glaciers of the Central-Western Asturian Mountains. in Iberia, Land of Glaciers 265–288 (Elsevier, 2022). doi: 10.1016/B978-0-12-821941-6.00013-X
168. Ruszkiczay-Rüdiger, Z. et al. Last deglaciation in the central Balkan Peninsula: Geochronological evidence from the Jablanica Mt. (North Macedonia). Geomorphology 351, 106985 (2020). doi: 10.1016/j.geomorph.2019.106985
169. Ruszkiczay-Rüdiger, Z. et al. Late Pleistocene glacial advances, equilibrium-line altitude changes and paleoclimate in the Jakupica Mts (North Macedonia). CATENA 216, 106383 (2022). doi: 10.1016/j.catena.2022.106383
170. Ruszkiczay-Rüdiger, Z., Kern, Z., Urdea, P., Madarász, B. & Braucher, R. Limited glacial erosion during the last glaciation in mid-latitude cirques (Retezat Mts, Southern Carpathians, Romania). Geomorphology 384, 107719 (2021). doi: 10.1016/j.geomorph.2021.107719
171. Santos-González, J., Redondo-Vega, J. M., Celis, A. G., González-Gutiérrez, R. B. & Gómez-Villar, A. The glaciers of the Leonese Cantabrian Mountains. in Iberia, Land of Glaciers 289–314 (Elsevier, 2022). doi: 10.1016/B978-0-12-821941-6.00014-1
172. Sarıkaya, M. A., Çiner, A. & Zreda, M. Quaternary Glaciations of Turkey. in Developments in Quaternary Sciences vol. 15 393–403 (Elsevier, 2011). doi: 10.1016/B978-0-444-53447-7.00030-1
173. Sawagaki, T. et al. Late Quaternary glaciations in Japan. in Developments in Quaternary Sciences vol. 2 217–225 (Elsevier, 2004). doi: 10.1016/S1571-0866(04)80127-1
174. Schlüchter, C. The Swiss glacial record – a schematic summary. in Developments in Quaternary Sciences vol. 2 413–418 (Elsevier, 2004). doi: 10.1016/S1571-0866(04)80092-7
175. Seif, A. Equilibrium-line altitudes of Late Quaternary glaciers in the Oshtorankuh Mountain, Iran. Quaternary International 374, 126–143 (2015). doi: 10.1016/j.quaint.2015.02.063
176. Serra, E., Valla, P. G., Gribenski, N., Carcaillet, J. & Deline, P. Post-LGM glacial and geomorphic evolution of the Dora Baltea valley (western Italian Alps). Quaternary Science Reviews 282, 107446 (2022). doi: 10.1016/j.quascirev.2022.107446
177. Serrano, E., Gómez-Lende, M. & González-Amuchastegui, M. J. The glaciers of the eastern massifs of Cantabria, the Burgos Mountains and the Basque Country. in Iberia, Land of Glaciers 157–178 (Elsevier, 2022). doi: 10.1016/B978-0-12-821941-6.00008-6
178. Serrano, E., Gómez-Lende, M. & Pisabarro, A. The glaciers of the western massifs of Cantabria. in Iberia, Land of Glaciers 201–219 (Elsevier, 2022). doi: 10.1016/B978-0-12-821941-6.00010-4
179. Serrano, E., González-1ba, J. J., Pellitero, R. & Gómez-Lende, M. Quaternary glacial history of the Cantabrian Mountains of northern Spain: a new synthesis. SP 433, 55–85 (2017). doi: 10.1144/SP433.8
180. Sharp, R. P. Pleistocene glaciation in the Trinity Alps of northern California. American Journal of Science 258, 305–340 (1960). doi: 10.2475/ajs.258.5.305
181. Sharp, R. P., Allen, C. R. & Meier, M. F. Pleistocene glaciers on southern California mountains. American Journal of Science 257, 81–94 (1959). doi: 10.2475/ajs.257.2.81
182. Sheinkman, V. S. Quaternary glaciation in the High Mountains of Central and North-east Asia. in Developments in Quaternary Sciences vol. 2 325–335 (Elsevier, 2004). doi: 10.1016/S1571-0866(04)80138-6
183. Silkwood, J. T. & United States Forest Service Northern Region. Glacial Lake Missoula and the Channeled Scabland: A Digital Portrait of Landforms of the Last Ice Age, Washington, Oregon, Northern Idaho, and Western Montana. (1998). doi:
184. Şimşek, M., Öztürk, M. Z., Yeşilyurt, S. & Utlu, M. Morphometric characteristics and paleogeographic implication of glacial cirques in Eastern Black Sea Mountains (Türkiye). Geomorphology 441, 108889 (2023). doi: 10.1016/j.geomorph.2023.108889
185. Soto, V., Delgado-Granados, H., Welsh, C. M. & Yoshikawa, K. Glacial reconstruction and periglacial dynamics at the end of Late Pleistocene on the surface of Cofre de Perote volcano, México: a climatological retrospective. J. Mt. Sci. 20, 2453–2467 (2023). doi: 10.1007/s11629-023-8230-3
186. Stansell, N. D., Polissar, P. J. & Abbott, M. B. Last glacial maximum equilibrium-line altitude and paleo-temperature reconstructions for the Cordillera de Mérida, Venezuelan Andes. Quaternary Research 67, 115–127 (2007). doi: 10.1016/j.yqres.2006.07.005
187. Stauch, G. & Lehmkuhl, F. Extent and Timing of Quaternary Glaciations in the Verkhoyansk Mountains. in Developments in Quaternary Sciences vol. 15 877–881 (Elsevier, 2011). doi: 10.1016/B978-0-444-53447-7.00064-7
188. Sugden, D. E. & Clapperton, C. M. The Maximum Ice Extent on Island Groups in the Scotia Sea, Antarctica. Quat. res. 7, 268–282 (1977). doi: 10.1016/0033-5894(77)90041-2
189. Suggate, R. P. South Island, New Zealand; ice advances and marine shorelines. in Developments in Quaternary Sciences vol. 2 285–291 (Elsevier, 2004). doi: 10.1016/S1571-0866(04)80134-9
190. Umer, M., Kebede, S. & Osmaston, H. Quaternary glacial activity on the Ethiopian mountains. in Developments in Quaternary Sciences vol. 2 171–174 (Elsevier, 2004). doi: 10.1016/S1571-0866(04)80122-2
191. Urdea, P., Onaca, A., Ardelean, F. & Ardelean, M. New Evidence on the Quaternary Glaciation in the Romanian Carpathians. in Developments in Quaternary Sciences vol. 15 305–322 (Elsevier, 2011). doi: 10.1016/B978-0-444-53447-7.00024-6
192. Valcarcel, M. & Pérez-Alberti, A. The glaciers in Western Galicia. in Iberia, Land of Glaciers 353–373 (Elsevier, 2022). doi: 10.1016/B978-0-12-821941-6.00017-7
193. Van Husen, D. Quaternary glaciations in Austria. in Developments in Quaternary Sciences vol. 2 1–13 (Elsevier, 2004). doi: 10.1016/S1571-0866(04)80051-4
194. VanSistine, P., Madole, R. F. & Michael, J. A. Data Release - Pleistocene Glaciation in the Upper Platte River Drainage Basin, Colorado. U.S. Geological Survey https://doi.org/10.5066/P9ROZWAZ (2022). doi: 10.5066/P9ROZWAZ
195. Vázquez-Selem, L. & Heine, K. Late Quaternary Glaciation in Mexico. in Developments in Quaternary Sciences vol. 15 849–861 (Elsevier, 2011). doi: 10.1016/B978-0-444-53447-7.00061-1
196. Vieira, G. et al. Penultimate Glacial Cycle glacier extent in the Iberian Peninsula: New evidence from the Serra da Estrela (Central System, Portugal). Geomorphology 388, 107781 (2021). doi: 10.1016/j.geomorph.2021.107781
197. Walter, L. M. Reconstructions of late pleistocene mountain glacier equilibrium line altitudes and paleoclimate in the great basin. (North Dakota State University, North Dakota, 2022). doi:
198. Walther, M. et al. Glaciers, Permafrost and Lake Levels at the Tsengel Khairkhan Massif, Mongolian Altai, During the Late Pleistocene and Holocene. Geosciences 7, 73 (2017). doi: 10.3390/geosciences7030073
199. Woodward, J. C. & Hughes, P. D. Glaciation in Greece. in Developments in Quaternary Sciences vol. 15 175–198 (Elsevier, 2011). doi: 10.1016/B978-0-444-53447-7.00015-5
200. Woodward, J. C. & Hughes, P. D. Glaciation in Greece. in Developments in Quaternary Sciences vol. 15 175–198 (Elsevier, 2011). doi: 10.1130/2020.2548(08)
201. Wright, H. E. & , Jr. Pleistocene glaciation in Kurdistan. E&G Quaternary Sci. J. 12, 131–164 (1962). doi: 10.3285/eg.12.1.12
202. Wright, H. E. Pleistocene Glaciation of Iraq. in Developments in Quaternary Sciences vol. 2 215–216 (Elsevier, 2004). doi: 10.1016/S1571-0866(04)80126-X
203. Xu, X., Wang, L., Yang, J. Last Glacial Maximum climate inferences from integrated reconstruction of glacier equilibrium-line altitude for the head of the Urumqi River, Tianshan Mountains. Quaternary International 218, 3–12 (2010). doi: 10.1016/j.quaint.2009.11.027
204. Yao, J., Chao-lu, Y., DanDan, X. & Nan, Y. Reconstruction of Palaeoglacier in the Qugaqie valley in Nyainqêntanglha Range. IOP Conf. Ser.: Earth Environ. Sci. 671, 012015 (2021). doi: 10.1088/1755-1315/671/1/012015
205. Zamoruyev, V. Quaternary glaciation of north-eastern Asia. in Developments in Quaternary Sciences vol. 2 321–323 (Elsevier, 2004). doi: 10.1016/S1571-0866(04)80137-4
206. Zasadni, J., Kłapyta, P. The Tatra Mountains during the Last Glacial Maximum. Journal of Maps 10, 440–456 (2014). doi: 10.1080/17445647.2014.885854
207. Žebre, M. & Stepišnik, U. Reconstruction of Late Pleistocene glaciers on Mount Lovćen, Montenegro. Quaternary International 353, 225–235 (2014). doi: 10.1016/j.quaint.2014.05.006
208. Žebre, M., Sarıkaya, M. A., Stepišnik, U., Yıldırım, C. & Çiner, A. First 36Cl cosmogenic moraine geochronology of the Dinaric mountain karst: Velež and Crvanj Mountains of Bosnia and Herzegovina. Quaternary Science Reviews 208, 54–75 (2019). doi: 10.1016/j.quascirev.2019.02.002
209. Zhou, S., Li, J., Zhao, J., Wang, J. & Zheng, J. Quaternary Glaciations. in Developments in Quaternary Sciences vol. 15 981–1002 (Elsevier, 2011). doi: 10.1016/B978-0-444-53447-7.00070-2
